# Supraphysiologic control over HIV-1 replication mediated by CD8 T cells expressing a re-engineered CD4-based chimeric antigen receptor

**DOI:** 10.1371/journal.ppat.1006613

**Published:** 2017-10-12

**Authors:** Rachel S. Leibman, Max W. Richardson, Christoph T. Ellebrecht, Colby R. Maldini, Joshua A. Glover, Anthony J. Secreto, Irina Kulikovskaya, Simon F. Lacey, Sarah R. Akkina, Yanjie Yi, Farida Shaheen, Jianbin Wang, Keith A. Dufendach, Michael C. Holmes, Ronald G. Collman, Aimee S. Payne, James L. Riley

**Affiliations:** 1 Department of Microbiology, Perelman School of Medicine, University of Pennsylvania, Philadelphia, Pennsylvania, United States of America; 2 Center for Cellular Immunotherapies, Perelman School of Medicine, University of Pennsylvania, Philadelphia, Pennsylvania, United States of America; 3 Department of Dermatology, Perelman School of Medicine, University of Pennsylvania, Philadelphia, Pennsylvania, United States of America; 4 Department of Medicine and Center for AIDS Research, Perelman School of Medicine, University of Pennsylvania, Philadelphia, Pennsylvania, United States of America; 5 Sangamo BioSciences Inc., Richmond, California, United States of America; Vaccine Research Center, UNITED STATES

## Abstract

HIV is adept at avoiding naturally generated T cell responses; therefore, there is a need to develop HIV-specific T cells with greater potency for use in HIV cure strategies. Starting with a CD4-based chimeric antigen receptor (CAR) that was previously used without toxicity in clinical trials, we optimized the vector backbone, promoter, HIV targeting moiety, and transmembrane and signaling domains to determine which components augmented the ability of T cells to control HIV replication. This re-engineered CAR was at least 50-fold more potent *in vitro* at controlling HIV replication than the original CD4 CAR, or a TCR-based approach, and substantially better than broadly neutralizing antibody-based CARs. A humanized mouse model of HIV infection demonstrated that T cells expressing optimized CARs were superior at expanding in response to antigen, protecting CD4 T cells from infection, and reducing viral loads compared to T cells expressing the original, clinical trial CAR. Moreover, in a humanized mouse model of HIV treatment, CD4 CAR T cells containing the 4-1BB costimulatory domain controlled HIV spread after ART removal better than analogous CAR T cells containing the CD28 costimulatory domain. Together, these data indicate that potent HIV-specific T cells can be generated using improved CAR design and that CAR T cells could be important components of an HIV cure strategy.

## Author summary

Conventional T cells rarely provide durable control over HIV replication. Chimeric antigen receptors (CARs) alter how T cells recognize infected T cells, bypassing many of HIV’s immune escape mechanisms. Therefore, we hypothesized that T cells engineered to express these receptors would be more potent (supraphysiologic) and would be able to control HIV infection at much lower effector to target ratios than conventional T cells. Starting with a CAR that entered clinical trials but did not reliably reduce measures of HIV in patients, we systematically optimized the expression, structure, and activation components to increase its functionality. CD8 T cells expressing this re-engineered CAR were extremely potent at suppressing HIV *in vitro*, exhibiting greater than 50-fold more potency in the ability to prevent viral spread than the original CAR construct or an HIV-specific TCR approach. Importantly, in a humanized mouse model of HIV infection, these optimized CAR T cells could protect CD4 T cells from HIV-mediated depletion and could significantly delay viral rebound after the cessation of ART. These data provide optimism that a re-engineered HIV-specific CAR may be able to provide durable control of HIV replication in patients.

## Introduction

It is well established that T cells play an important role in controlling HIV-1 (HIV) replication, and that HIV-infected individuals develop robust HIV-specific T cell responses [1, 2]. However, HIV evades the endogenous T cell-mediated immune response by altering key residues required for T cell recognition and downregulating class I major histocompatibility complexes (MHC) [3, 4]. Additionally, HIV-mediated depletion of HIV-specific CD4 T helper cells and the chronic persistence of the virus functionally impair HIV-specific CD8 T cells so that, in most individuals, T cells are unable to control HIV replication [5]. Although antiretroviral therapy (ART) can suppress HIV replication by many orders of magnitude, it fails to eliminate the virus, forcing HIV-infected individuals to be treated by ART regimens for the rest of their lives. ART also reduces the number of HIV-specific T cells present, due to a massive reduction in HIV antigen, and the T cells that remain often have functional defects [6, 7]. Thus, at the time of ART removal when the number of HIV producing cells is minimal, the resident HIV-specific T cell immune response is ill-equipped to control the re-emerging infection and uniformly fails, with viral loads returning to patient-specific set point within weeks after ART removal [8]. As a result, we and others have postulated that instead of relying on the endogenous immune response to control HIV replication as part of an HIV cure strategy, the introduction of a potent engineered immune response designed to overcome HIV’s escape mechanisms will be required to provide durable control HIV in the absence of ART [9–11].

Previous adoptive cell therapy (ACT) trials have demonstrated that simply reinfusing expanded patient T cells does not result in durable control over HIV replication [9]. Thus, both the quality and quantity of infused HIV-specific T cells must be enhanced for a sustained therapeutic benefit. Attempts to manufacture better T cells for ACT have recently been made through antiviral transgenes, coreceptor editing, and redirecting T cells with HIV-specific T cell receptors (TCRs) or chimeric antigen receptors (CARs) [9, 12]. CARs consist of an extracellular antigen binding domain fused to intracellular T cell activation domains [13]. These synthetic receptors can redirect T cells to recognize viral proteins independent of antigen processing, TCR, and MHC. CARs targeting CD19 have revolutionized the treatment of leukemia and lymphomas through their ability to persist and maintain durable anti-tumor effects *in vivo* [14, 15]. Of note, the first CAR to enter human trials redirected T cells to target the GP120 region of the HIV Envelope (Env) glycoprotein [16–18]. This was achieved by fusing the extracellular and transmembrane domains of CD4, the cellular receptor for HIV, to the CD3-zeta cytoplasmic region (CD4-zeta). HIV-specific cytotoxicity was established *in vitro*, and safety was demonstrated *in vivo*, with transient reductions in HIV RNA, DNA, and quantitative HIV outgrowth assays [16, 17, 19–21]. A long-term follow up study determined the half-life of the CAR-modified cells to be over 16 years, with CAR expression up to 10 years post infusion and no serious adverse events [22]. However, the CD4-zeta CAR did not lead to sustained reductions in viral loads or the viral reservoir, and the clinical data were not sufficiently promising to warrant additional development.

Subsequent to the HIV CAR clinical trials, a number of advances have been made in CAR design for optimal antitumor responses. Xenograft models and clinical trials have established that costimulation augments function, proliferation, and survival *in vivo* [23–25]. In addition, CAR structural and signaling domains have been found to greatly impact T cell function and susceptibility to exhaustion [26, 27]. For example, it was recently demonstrated that CAR costimulatory domains influence T cell metabolic and phenotypic profiles, with 4-1BB promoting a central memory phenotype and CD28 promoting an effector memory phenotype [28]. While there has recently been a renewed interest in utilizing CARs to control HIV, including efforts to increase CAR T cell function and survival, a systematic optimization of CAR T cell cytotoxicity was lacking [29, 30]. We sought to apply the lessons learned from engineering CARs for hematologic malignancies to optimize the CD4 CAR for superior control over HIV replication. We demonstrate here that changing the CAR expression vector, promoter, transmembrane, and costimulatory domains improved control over HIV *in vitro* by over 50-fold. *In vivo*, humanized mice engrafted with T cells expressing these optimized vectors had significantly higher CD4 T cell counts, greater CAR^+^ CD8 T cell proliferation after HIV infection, and 90% less HIV RNA, compared to mice that received T cells transduced with the clinical trial CD4-zeta CAR. An HIV treatment model demonstrated superior control over HIV replication by 4-1BB containing CARs, compared to similar CARs containing the CD28 costimulatory domain. These data provide a compelling reason to revisit human clinical trials with CD4 CARs that have been optimized for control over HIV *in vivo* for use in HIV cure studies.

## Results

### Lentiviral backbone augments CAR expression and control over HIV replication

**Fig 1:**
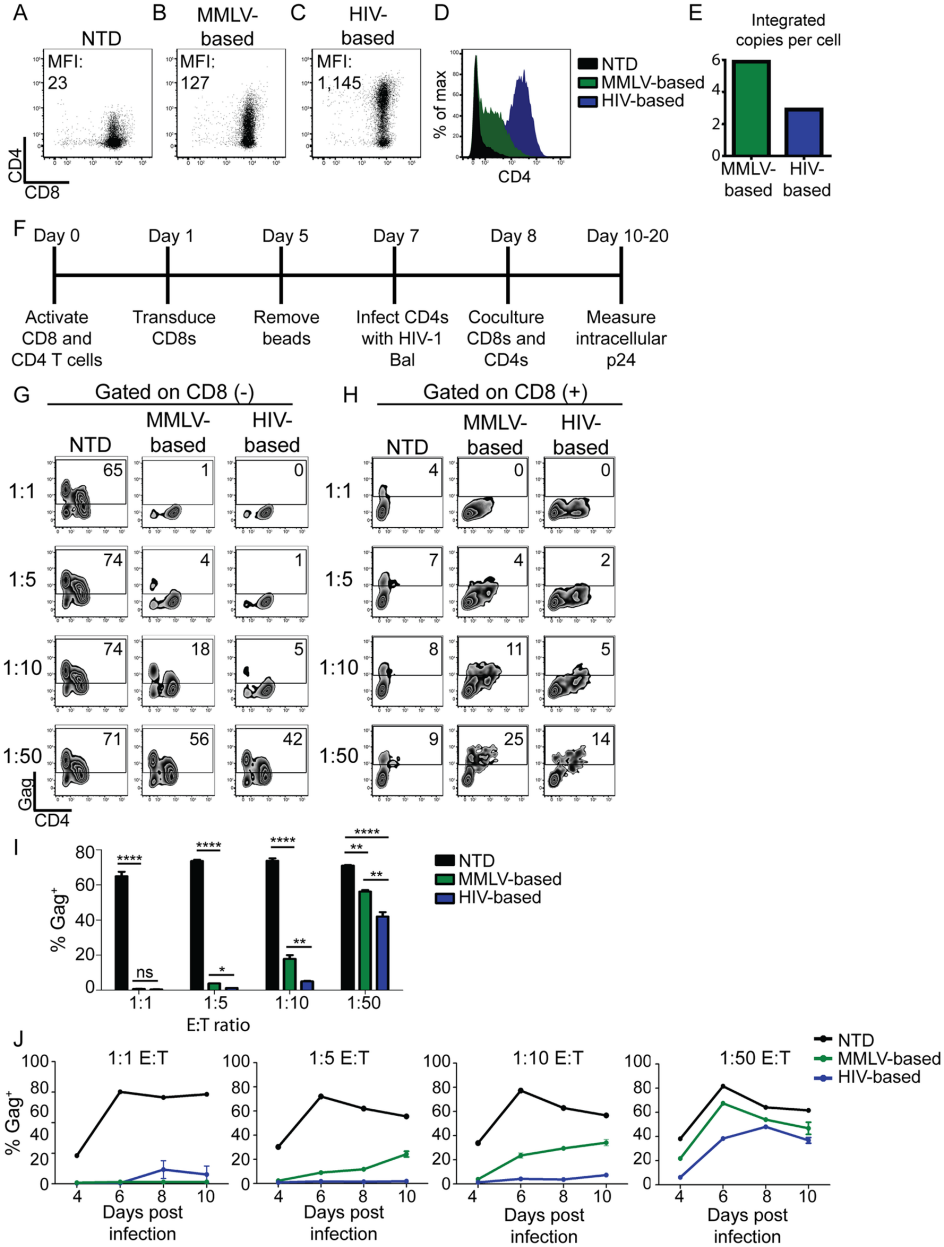
Lentiviral backbone augments CAR expression and control over HIV replication. **(A-D)** Primary human CD8 T cells were activated with αCD3/αCD28 coated beads and were either left **(A)** nontransduced (NTD), **(B)** transduced with the original MMLV-based CD4 CAR, or **(C)** transduced with the same CAR placed in a HIV-based lentiviral vector, both driven by the PGK promoter. After eight days T cells were stained for CD4 and CD8 by flow cytometry. Median fluorescence intensity (MFI) is indicated on each graph. (**D)** Overlying histograms of the data shown in **(A-C). (E)** Eight days post activation, qPCR was performed and the number of integrated vector copies per cell was calculated. (**F**) Schematic of experimental design to study the control over HIV replication by T cells expressing HIV-specific CARs. Briefly, following activation with αCD3/αCD28 coated beads, CD4 T cells were infected with HIV Bal, and 24 hours later the indicated CD8 T cells were mixed at the indicated effector to target (E:T) ratios. After 7 days of co-culture, the expression of surface CD4, CD8, and intracellular Gag was measured by flow cytometry. **(G)** Intracellular Gag staining on CD8 negative cells, and **(H)** Intracellular Gag staining on CD8 positive cells. **(I)** Summary data for a single experiment, performed in triplicate, gating on the CD8 negative cells. Error bars indicate standard error of the mean (SEM). Significance was detected using a 1-way ANOVA test, stratifying based on the E:T ratio (p values: ns >0.05, *<0.05, **<0.01, ***<0.0001). This data is representative of three independent experiments. S14 Fig shows each of the 3 independent experiments. (**J)** Measurement of levels of intracellular Gag in CD8 negative T cells over the time course of an experiment. Each graph represents a different E:T ratio. Error bars indicate SEM (n = 3).

Preclinical studies testing the original CD4-zeta CAR showed that T cells expressing this construct had equivalent antiviral activity to naturally generated HIV-specific T cells [21]. We hypothesized that the reason these clinical trials failed to demonstrate durable clinical responses was that the T cells used in these trials were no more potent than the endogenous HIV-specific T cell response. Therefore, we sought to optimize the CD4-zeta CAR based on lessons learned from cancer-specific CARs, in order to augment control over HIV replication [31]. To do this, we optimized each component of the CAR in a step-by-step manner. We first addressed to what extent the vector backbone contributes to the ability of CAR^+^ CD8 T cells to control HIV. The original CD4-zeta CAR was expressed by a murine retroviral vector (MMLV-based). Since MMLV-based vectors target promoter regions and lentiviral vectors (HIV-based) integrate preferentially into open reading frames, we reasoned that lentiviral vectors would result in higher expression than MMLV-based vectors [32]. We generated the MMLV clinical trial construct and an analogous lentiviral vector, both with the PGK promoter, and transduced primary human CD8 T cells. Lentiviral transduction consistently resulted in a ~10-fold higher median fluorescence intensity (MFI) of CAR expression compared to MMLV retrovirus (Fig 1A–1D). To determine whether the higher transgene expression was the result of more vector integrations per cell, we measured the integrated vector copy number in the different T cell populations using a previously established assay [22]. We found that the MMLV-based vector had almost twice the number of integrations compared to the HIV-based vector (Fig 1E), indicating that the higher transgene expression of the HIV-based vector is due to its intrinsic properties.

A co-culture assay was used to compare the different CARs in terms of their ability to control HIV replication (Fig 1F). In this assay, HIV-infected CD4 T cells were cultured with either nontransduced (NTD) CD8 T cells or CAR transduced CD8 T cells, and the ability of the effector CD8 T cells to control HIV spread was measured over 7–14 days. To distinguish between HIV spread throughout the CD4 T cells and infection of the CD4 CAR^+^ CD8 T cells, we gated separately on the CD8 negative and the CD8 positive T cells, and then analyzed intracellular p24 (Gag) in CD4 T cells and CD8 T cells, respectively (see gating strategy in S1 Fig). High levels of HIV replication were observed in CD4 T cells co-cultured with NTD CD8s (Fig 1G). In contrast, CD8 T cells transduced with either a MMLV-based or HIV-based CAR vector were able to control HIV replication at a 1:1 E:T ratio, similar to what was observed in previous studies of this strategy [19–21]. However, upon diluting the CAR transduced CD8 T cells to lower E:T ratios, T cells transduced with the HIV-based vector were superior at controlling HIV replication over time (Fig 1G–1J). Ultimately, neither population of transduced CD4 CAR CD8 T cells could control HIV spread at a 1:50 E:T ratio.

In contrast to recent reports that CD4 CAR transduced CD8 T cells are susceptible to infection by cell-free virus [29, 30], we were only able detect intracellular Gag in CAR^+^ CD8 T cells after diluting to low E:T ratios with HIV-infected CD4 T cells (Fig 1H, S2 Fig). Of note, there was a small proportion of nontransduced CD8 T cells that stained positively for Gag, regardless of the E:T ratio. This was likely the result of the ability of CD8 T cells to transiently express CD4 after activation [33–36]. This data highlights the complex relationship between CD4 CAR expression and susceptibility to HIV infection. At high E:T ratios the CAR^+^ CD8 T cells are able to fully suppress HIV replication, and thus they and the co-cultured CD4 T cells are protected from HIV infection. However, at low E:T ratios, the CAR^+^ CD8 T cells are no longer able to suppress HIV replication, and the virus is able to spread throughout both populations of cells (Fig 1H).

### EF1α promoter and CD8α transmembrane domains improve CAR expression and control over HIV

**Fig 2:**
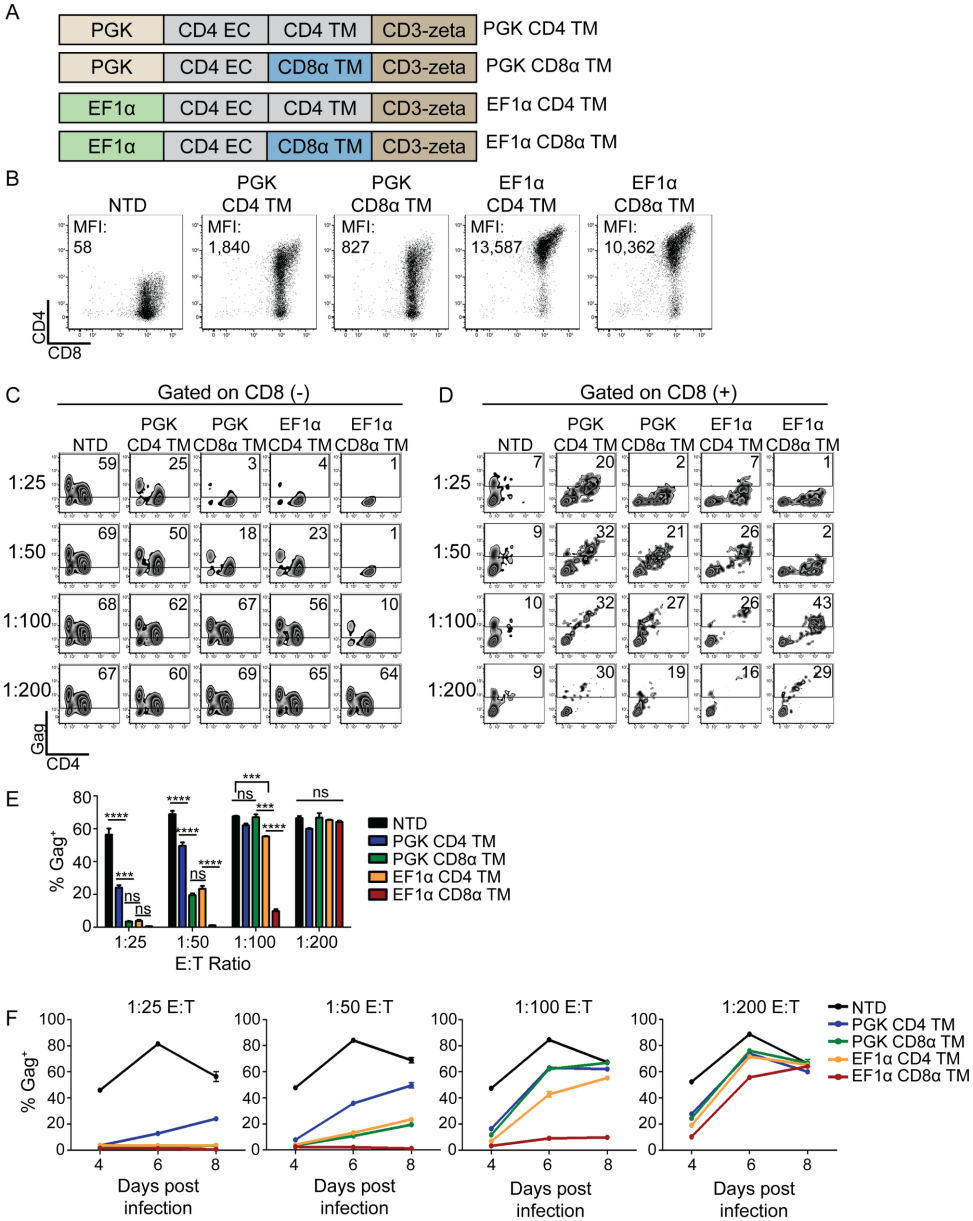
EF1α promoter and CD8α transmembrane domains improve CAR expression and control over HIV. **(A)** Schematic of the constructs compared in this figure. (**B)** CD4 CAR expression 8 days after activation. Median fluorescence intensity (MFI) is indicated on each graph. (**C**) Intracellular Gag staining on day 7 of co-culture, for CD8 negative T cells and **(D)** for CD8 positive T cells. **(E)** Summary data for a single experiment, performed in triplicate, gating on the CD8 negative cells. Error bars indicate SEM. Significance was detected using a 1-way ANOVA test, stratifying based on the E:T ratio (p values: ns >0.05, *<0.05, **<0.01, ***<0.0001). This data is representative of three independent experiments. S15 Fig shows each of the 3 independent experiments. (**F)** The levels of intracellular Gag in CD8 negative T cells over the time course of an experiment. Each graph represents a different E:T ratio. Error bars indicate SEM (n = 3).

Transgene expression in T cells wanes when driven by the PGK promoter as T cells rest down. In contrast, the EF1α promoter induces higher expression that is better sustained as T cells return to quiescence [23]. We hypothesized that the EF1α promoter might be beneficial in our system, since greater CAR MFI expression correlated with better control over HIV (Fig 1). Under the EF1α promoter, CAR expression MFI increased ~10-fold compared to the PGK promoter (Fig 2A and 2B). Next, we substituted the CD8α transmembrane (TM) domain in place of the CD4 TM domain to promote CAR dimerization, remove CD4 TM motifs targeted by HIV Vpu for downregulation, decrease homology to the HIV cellular receptor, and ultimately augment cytotoxicity [37–40]. Greater control over HIV replication was achieved by both modifications individually, and a combination of the two modifications led to complete control over HIV replication down to a 1:50 E:T ratio (Fig 2C and 2E). Substitution of the CD8α TM domain decreased infection of CAR CD8 T cells regardless of the promoter used at the 1:25 and 1:50 E:T ratios (Fig 2D). We observed similar results when examining the culture supernatants for p24 Gag (S3 Fig). However, as seen in Fig 1 and S2 Fig, the CAR^+^ CD8 T cells could be diluted to the point where they no longer controlled HIV infection and succumbed to infection themselves (Fig 2D). To ensure this was not an artifact of gating on a few CD8 T cells, we performed a larger scale experiment where at least 1x10^4^ CD8 T cells were collected per condition and the infection pattern was the same (S4A and S4B Fig). HIV rapidly downregulates CD4 expression in an HIV Nef dependent manner, and we wanted to determine whether the CD4 CAR construct was susceptible to downregulation by HIV infection. Therefore, we compared CD4 expression in HIV-infected primary human CD4 T cells and CD4 CAR transduced CD8 T cells (S4C Fig). We observed that CD4 CAR expression was maintained at a high level even in presence of robust HIV infection. This finding is consistent with previous findings that demonstrated a dileucine motif within the CD4 cytoplasmic tail is required for HIV Nef mediated downregulation [41] and since CD4 CAR does not have this motif, it is immune to HIV Nef downregulation. Thus, CD4 CAR T cells may still function even after being infected with HIV. Thus, altering the viral vector, promoter, and transmembrane domains afforded a 50-fold increase in potency over the clinical trial MMLV-based retrovirus, resulting in complete control over HIV replication at a 1:50 E:T ratio *in vitro* (Fig 2E and 2F).

### Re-directed T cells expressing a CD4 CAR are 100-fold more potent than re-directed T cells specific for B57-KF11

**Fig 3:**
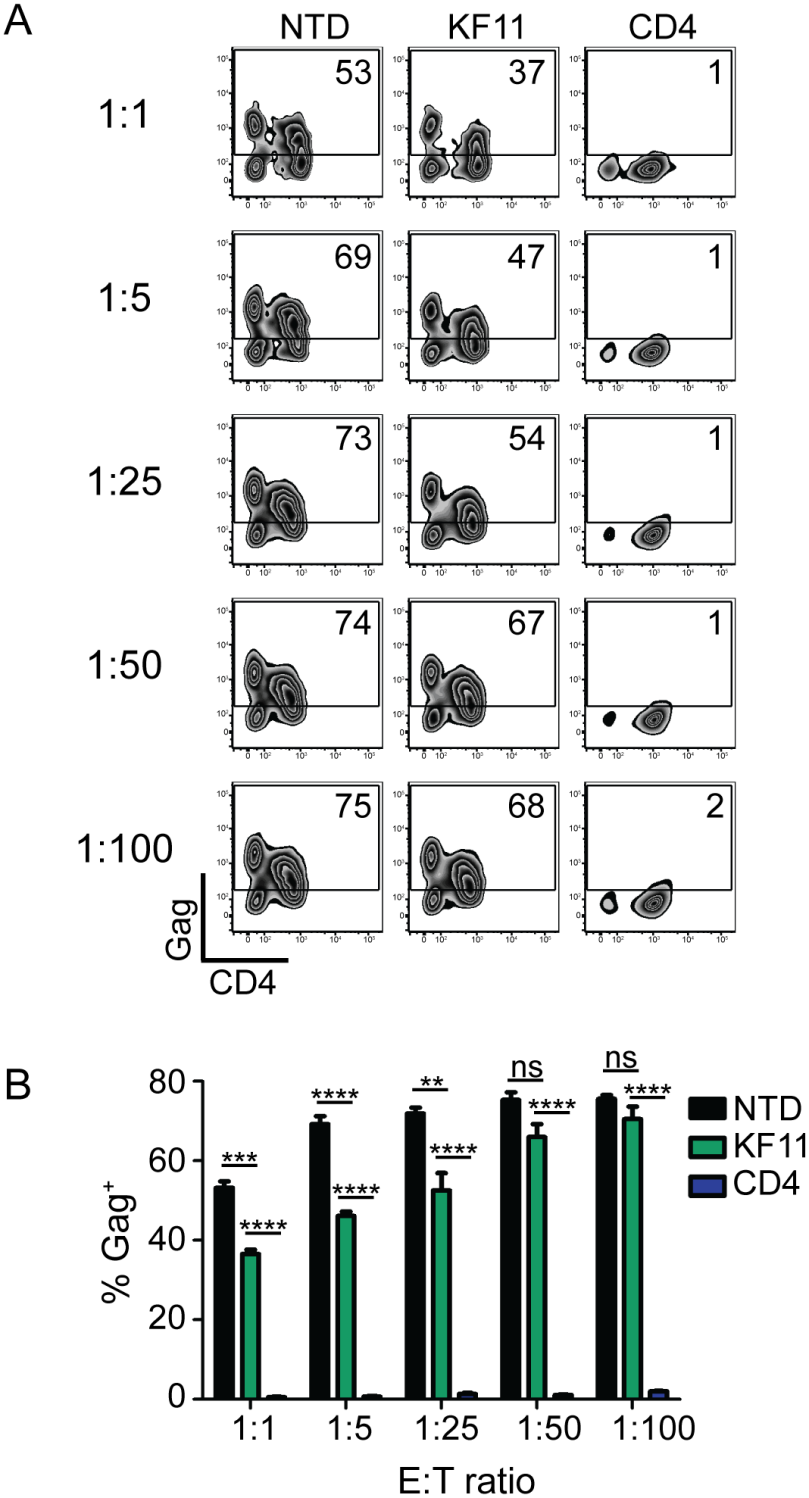
Re-directed T cells expressing a CD4 CAR are 100-fold more potent than re-directed T cells specific for B57-KF11. **(A)** Gag staining on day 6 of co-culture for CD8 negative T cells. **(B)** Summary data for a single experiment performed in triplicate, gating on the CD8 negative T cells. Error bars indicate SEM. Significance was detected using a 1-way ANOVA test, stratifying based on the E:T ratio (p values: ns >0.05, *<0.05, **<0.01, ***<0.0001). This data is representative of three independent experiments. S16 Fig shows each of the 3 independent experiments.

Elite controllers are rare individuals who control HIV replication in the absence of ART. Certain HLA alleles, such as HLA-B57, are overrepresented in these cohorts, suggesting that T cell responses play a key role in controlling their virus [42]. Therefore, we wished to determine whether T cells expressing a re-engineered CD4 CAR (with the EF1α promoter and CD8α TM) could control HIV replication better than T cells expressing a HLA-B57 restricted TCR that is associated with better control over HIV replication. To do this, we generated an analogous lentiviral vector that expressed the TCRα and TCRβ chain (obtained from B57-KF11 specific T cell clone generated from an elite controller generously provided by Xu Yu and Bruce Walker) linked by T2A sequence which when expressed in T cells conferred specificity for B57-KF11 epitope (HIV p24 Gag epitope KAFSPEVIPMF). To confirm these cells recognized Gag-expressing cells, we mixed them with target CD4 T cells from HLA-B57^+^ individuals that were transfected with Gag RNA, or Pol RNA as a negative control, and detected a robust Gag-specific cytokine response (S5B Fig). Next, we compared the ability of KF11 TCR versus CD4 CAR transduced CD8 T cells to limit HIV spread in HLA-B57^+^ CD4 T cells. While KF11 TCR-transduced CD8 T cells reduced HIV replication down to a 1:25 E:T ratio, complete control over HIV replication was never achieved (Fig 3). In contrast, the re-engineered CD4 CAR controlled HIV almost completely down to a 1:100 E:T ratio. Efforts to improve the efficacy of B57-KF11 transduced T cells could be achieved by finding higher avidity TCRs [43] or by approaches that force B57-KF11 TCR pairing. Nonetheless, these data suggest that the synthetic CD4 CAR approach is more potent than the natural TCR based approach and that the CD4 CAR approach will likely be more effective as a cellular therapy tool compared to T cells transduced with a patient-derived, natural TCR.

### CD4-based CARs control HIV more effectively than broadly neutralizing antibody-based CARs

**Fig 4:**
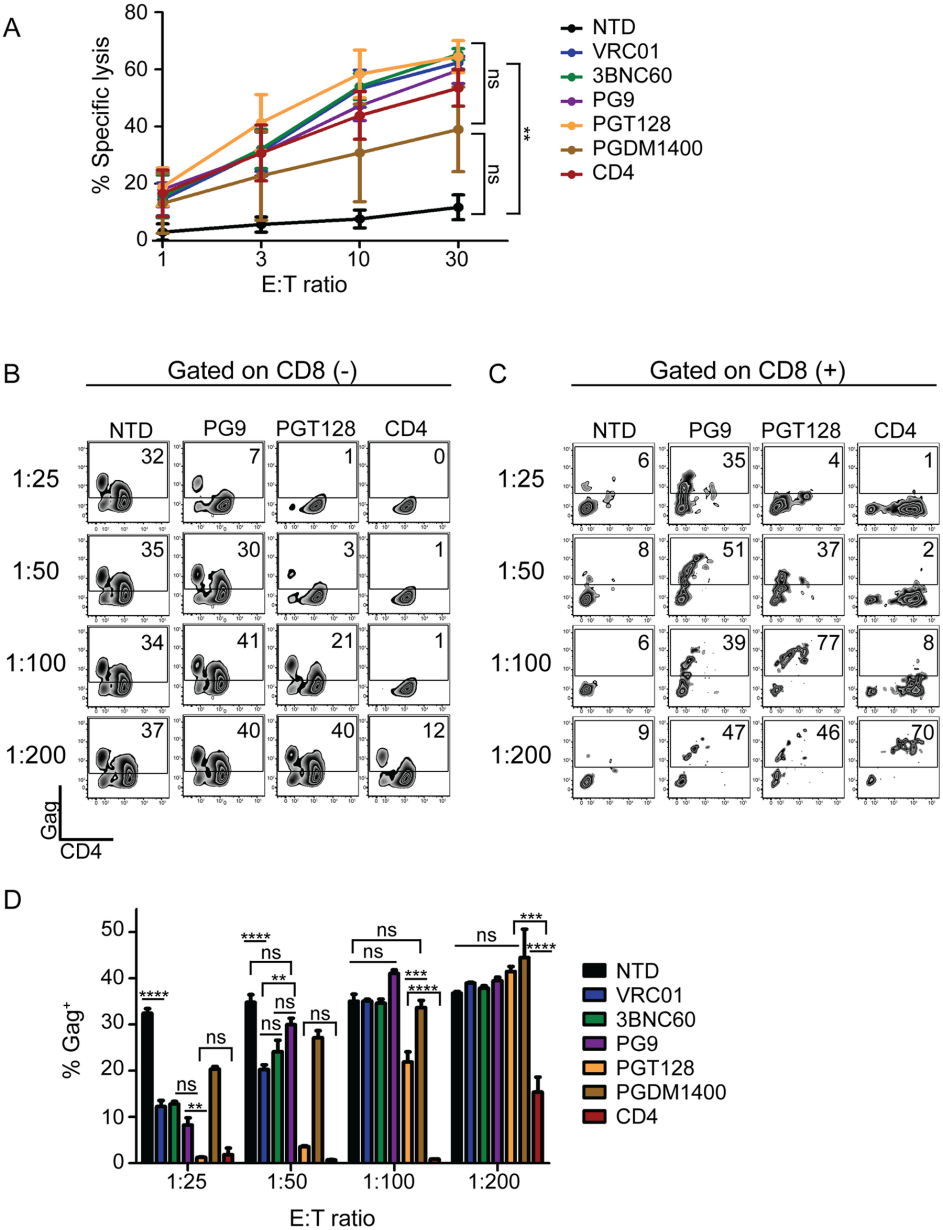
CD4-based CARs control HIV more effectively than broadly neutralizing antibody-based CARs. **(A)** Specific lysis of Cr^51^ labeled K562 target cells expressing HIV-1 YU2 GP160. Significance was detected using a 1-way ANOVA test on the 30:1 E:T ratio (p values: ns >0.05, *<0.05, **<0.01, ***<0.0001). Data plotted shows the average of three independent experiments. Error bars indicate SEM (n = 3). **(B)** Gag staining on day 6 of co-culture for CD8 negative T cells and **(C)** the CD8 positive T cells. The data from the best (PGT128) and one of the worst (PG9) scFv-based CARs are compared to the CD4 CAR here, but the complete construct comparison is presented in S6 Fig. (**D)** Summary data for a single experiment performed in triplicate, gating on the CD8 negative cells. Error bars indicate SEM. Significance was detected using a 1-way ANOVA test, stratifying based on the E:T ratio (p values: ns >0.05, *<0.05, **<0.01, ***<0.0001). Data is representative of three independent experiments. S17 Fig shows each of the 3 independent experiments.

The most commonly used CAR ectodomains are antibody-derived single chain variable fragments (scFvs) [13]. Over the past several years, a number of HIV-specific antibodies have been described that bind or neutralize HIV with high affinity and/or target a wide breadth of viruses [44]. We tested whether the use of scFvs to redirect T cells to HIV was superior to the use of CD4. In addition, we wanted to test whether CD8 T cells expressing scFv-based CARs were less susceptible to HIV infection than CD4-based CARs. A panel of scFvs derived from the VRC01, 3BNC60, PG9, PGT128, or PGDM1400 parental antibodies were generated due to their neutralization breadth and/or potency against HIV and cloned into the most effective CAR design identified in Fig 2, with the EF1α promoter and the CD8α TM domain [45, 46]. To determine that each scFv CAR had folded properly, could interact with Env, and promote CD8 T cell lysis, we measured specific lysis of chromium labeled K562 target cells expressing the Env protein from the HIV YU2 strain. All CARs were capable of lysing Env-expressing targets to a similar degree, indicating that these CARs recognized HIV Env (Fig 4A). Many of the scFv CARs consistently produced higher levels of intracellular cytokines in response to Env-expressing targets when compared to the CD4 CAR (S6A Fig). However, when co-cultured with HIV-infected CD4 T cells, the CD4 CAR controlled HIV better than all of the scFv-based CARs (Fig 4B and 4D and S6B Fig). Interestingly, the PGT128 CAR repeatedly controlled HIV better than the other scFvs tested, despite being less broad and less potent in neutralization studies than PGDM1400 [46]. Surprisingly, at low E:T ratios we detected high levels of intracellular Gag in the scFv CD8 T cells, similar to CD4 CAR^+^ T cells diluted to a 1:200 E:T (Fig 4C). This was not a byproduct of lentiviral transduction or generic CAR expression, as this was not seen for GFP-transduced or CD19 CAR-transduced cells (S6C Fig) and thus appears to depend on HIV binding ability, possibly concentrating the virus near the CAR-transduced cell membrane. Based on the *in vitro* superiority of the CD4 CAR against this limited scFv subset, and the inherent difficulty of HIV escaping from CD4 binding, we chose to pursue development of CD4-based CARs for *in vivo* testing.

### ICOS, CD27, and 4-1BB costimulation impair control of HIV replication *in vitro*

**Fig 5:**
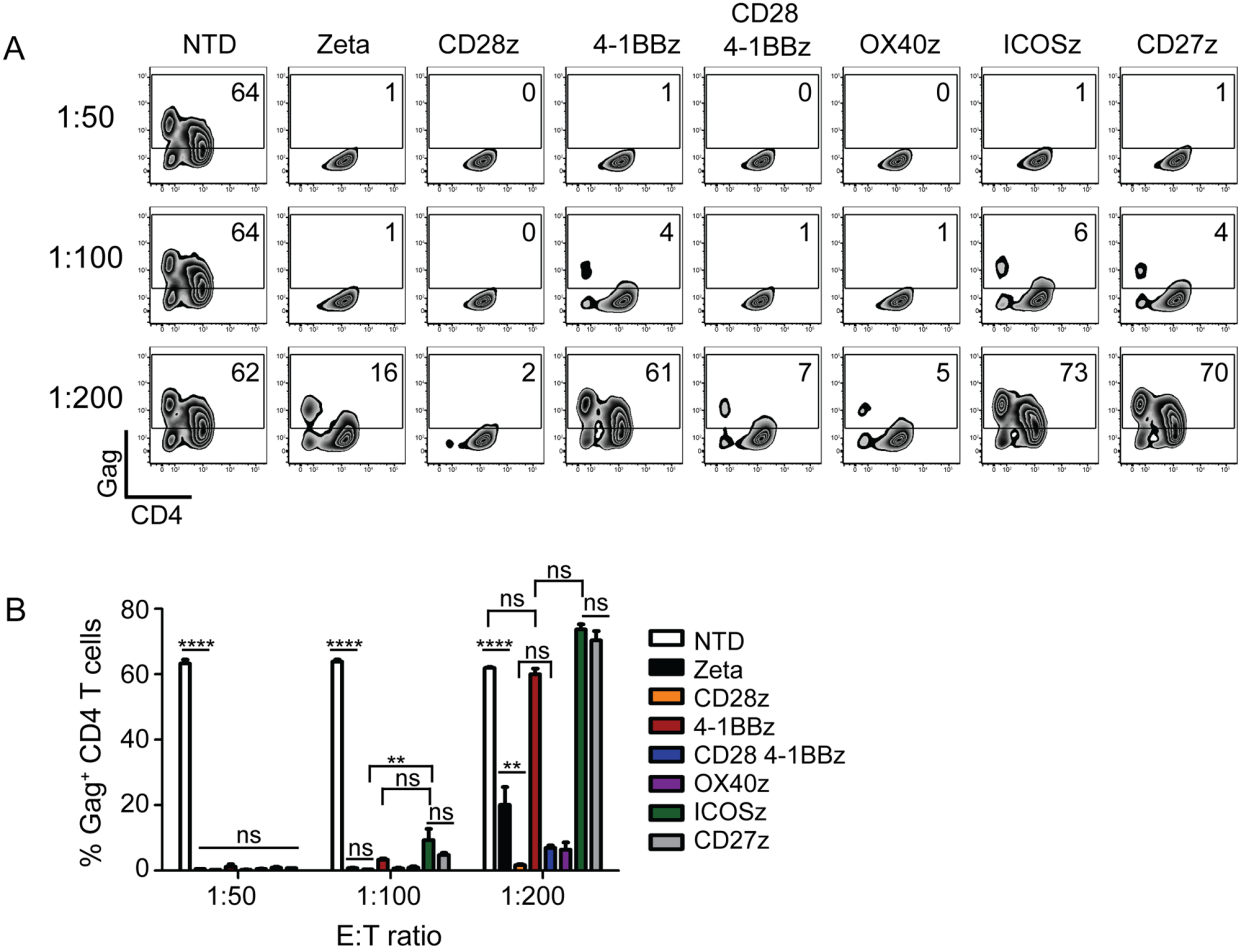
ICOS, CD27, and 4-1BB costimulation impair control of HIV replication *in vitro*. **(A)** Gag staining on day 9 of co-culture for CD8 negative T cells. **(B)** Summary data for a single experiment performed in triplicate, gated on the CD8 negative T cells. Error bars indicate SEM. Significance was detected using a 1-way ANOVA test, stratifying based on the E:T ratio (p values: ns >0.05, *<0.05, **<0.01, ***<0.0001). Data is representative of three independent experiments. S18 Fig shows each of the 3 independent experiments.

T cells require costimulatory signals for proliferation, effector function, and long-term survival [47]. Costimulatory domains, such as CD28 and 4-1BB, have been incorporated into recent CAR designs for durable CAR T cell responses *in vivo* [24]. In chronic HIV infection, T cell dysfunction and exhaustion have been well documented, and decreased CD28 and 4-1BB signaling impair cytolytic and effector function [7, 48, 49]. Therefore, we generated a panel of CD4 CARs that incorporated a variety of costimulatory domains in conjunction with the CD3-zeta domain, including CD28, 4-1BB, CD28+4-1BB, OX40, ICOS, or CD27 and tested their ability to control HIV infection *in vitro*. CD8 T cells expressing CARs that contained 4-1BB, CD27, or ICOS costimulation domains did not control HIV as effectively as T cells expressing CARs that expressed the other costimulatory domains, suggesting that these costimulatory pathways interfere with control over HIV replication (Fig 5). Regardless of the costimulatory domain, the control seen with these CD4 CARs was superior to the control seen with an HIV-specific TCR (Fig 3) or with scFv based CARs (Fig 4). While CD28 promoted better control over HIV *in vitro* compared to 4-1BB, discrepancies between the *in vitro* and *in vivo* activity of cancer-specific CARs containing 4-1BB had been reported [26, 28]. Thus, we were curious if this held true for HIV-specific CARs, and decided to further characterize the safety profile and *in vivo* efficacy of CD4 CARs expressing either CD28 or 4-1BB.

### CD4 CARs respond to Env^+^ cells and not MHC class II^+^ cells

**Fig 6:**
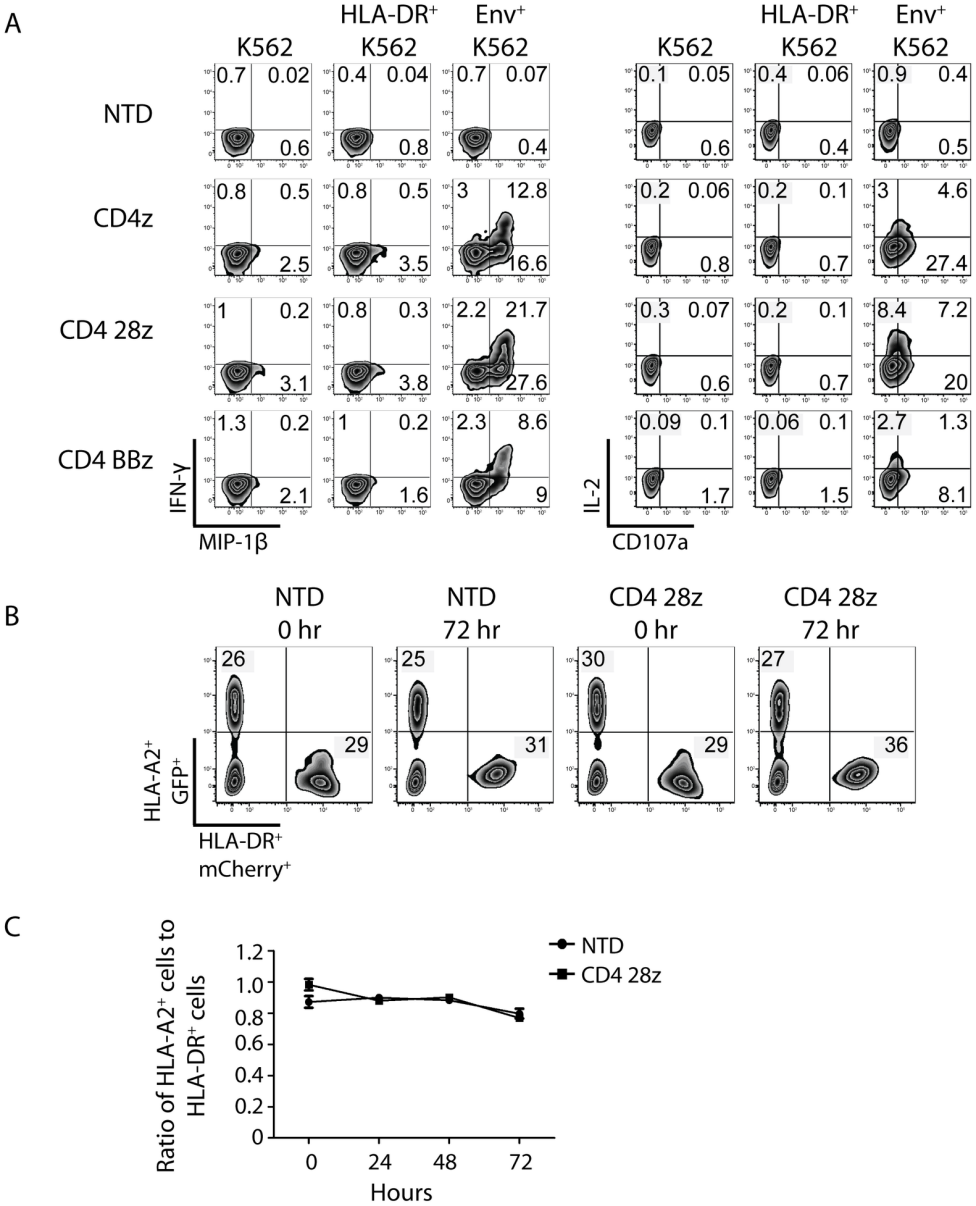
CD4 CARs respond to Env^+^ cells and not MHC class II^+^ cells. **(A)** Primary human CD8 T cells were activated with either left NTD or transduced with the indicated CD4 CARs. Two weeks post activation, the CD8 T cells were co-cultured for 6 hours at a 1:1 ratio with unmodified K562 cells, K562 cells expressing high levels of HLA-DR, or K562 cells expressing HIV-1 YU2 GP160. Intracellular IFNγ and MIP-1β expression is shown on the left, and intracellular IL-2 expression and CD107a surface mobilization is shown on the right. **(B)** A co-culture assay was designed to demonstrate that CD4 CAR^+^ CD8 T cells do not kill MHC class II-expressing target cells. Briefly, NTD or CD4 28z CAR transduced CD8 T cells from **(A)** were co-cultured with K562 cells expressing both HLA-A2 and GFP as well as K562 expressing both HLA-DR*0401 and mCherry at a 1:1:1 ratio. Flow cytometry measuring GFP and mCherry expression was performed immediately after mixing (0 hr) and after 3 days of co-culture (72 hr). **C)** Summary data for a single experiment performed in triplicate, measuring the ratio of HLA-A2/GFP-expressing cells to HLA-DR*0401/mCherry-expressing cells after 24, 48, and 72 hours of culture. Error bars indicate SEM. Data is representative of three independent experiments.

Preclinical data demonstrated that T cells expressing the clinical trial CD4-zeta CAR did not kill Raji cells, which express high levels of MHC class II, the low affinity ligand of CD4 [20]. However, we were concerned that our optimized, highly expressed CD4 CAR might recognize MHC class II expressing cells. To test this, we measured CD4 CAR transduced CD8 T cell responses against K562 cells stably expressing high levels of the HLA-DR*0401 allele (S7 Fig). CD8 T cells were transduced with optimized CD4 CARs containing CD3-zeta alone or with the 4-1BB and CD28 costimulatory domains and cultured with unmodified K562 target cells, HLA-DR*0401^+^ K562 cells, or HIV YU2 Env^+^ K562 cells as a positive control. CD4 CAR transduced CD8 T cells produced IL-2, CD107a, IFN-γ, and MIP-1β in response to HIV Env^+^ targets but not in response to HLA-DR^+^ or parental K562s, with the most robust production by CD28-containing CAR T cells (Fig 6A). A small MIP-1β signal was observed for all CARs mixed with parental or HLA-DR expressing targets that was not observed with NTD controls, likely due to some constitutive signaling observed in CAR T cells [50]. It has been shown that autocrine production of beta chemokines by CMV-specific T cells decreases CCR5 expression and protects these cells from HIV infection, so this low-level MIP-1β production may help protect the CAR^+^ CD8 T cells *in vivo [51]*. Importantly, no difference in cytokine or CD107a production was detected by CAR T cells cultured with parental versus MHC class II expressing cells, suggesting that our optimized CD4 CARs do not facilitate off-target recognition of MHC class II. Coculturing the optimized CD28-containing CAR with HLA-DR^+^ K562 cells over an extended period further confirmed the lack of off-target responses. We mixed HLA-DR^+^ K562 cells 1:1 with HLA-A2^+^ K562 cells, which served as a negative control, and did not see a change in the ratio of the two K562 populations over time (Fig 6B and 6C), suggesting that the re-engineered CAR will exhibit a similar safety profile in humans as the original CD4 CAR.

### Optimized CAR T cells control HIV replication better and expand to greater levels *in vivo* than first generation CAR T cells

**Fig 7:**
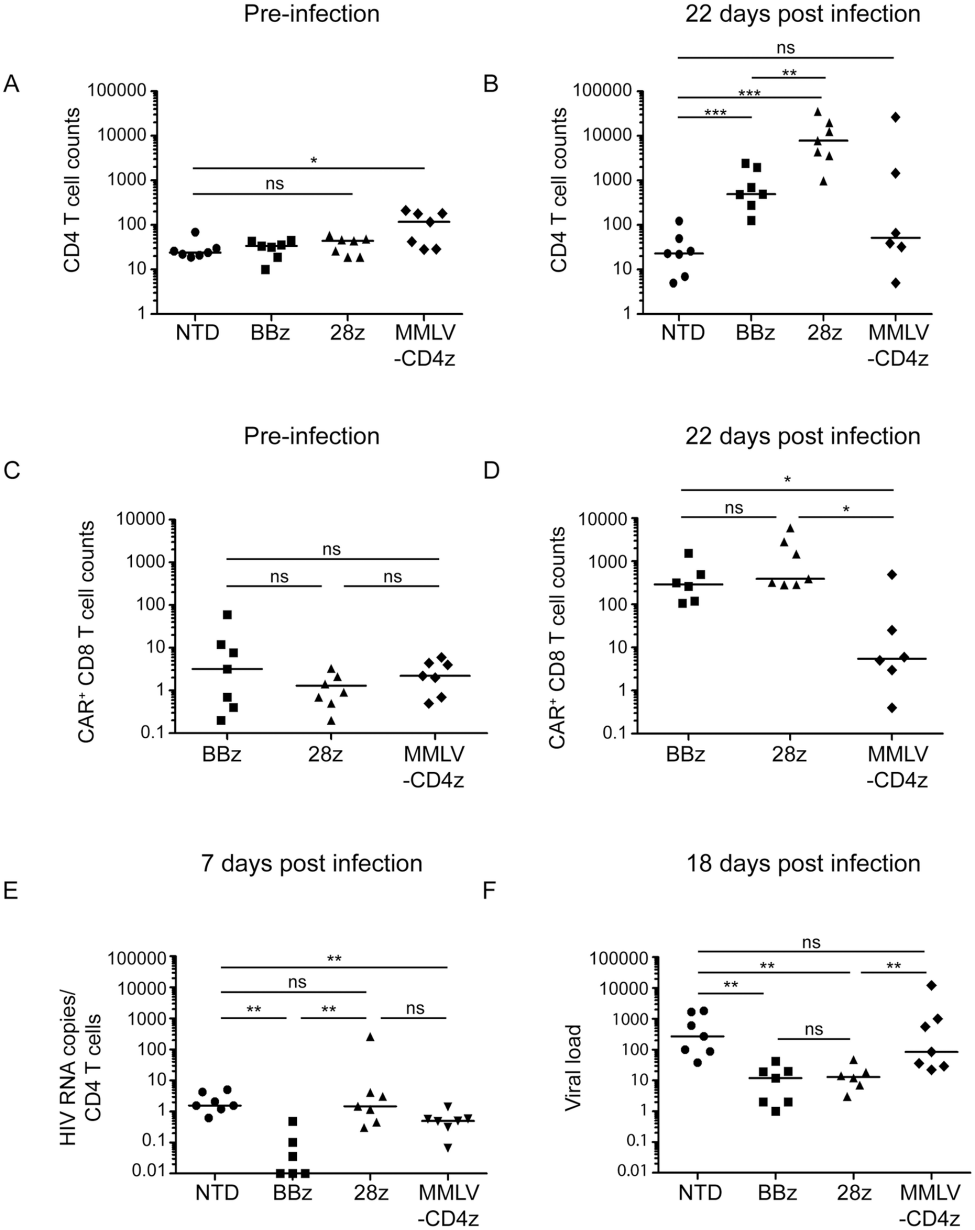
Optimized CAR T cells control HIV-1 replication better and expand to greater levels *in vivo* than first generation CAR T cells. Cohorts of NSG mice were infused with 8 million human CD4 T cells and 2 million human CD8 T cells (50% CAR transduction efficiency). CD8 T cells were either left NTD, transduced with optimized CD4 CARs containing either 4-1BB or CD28 intracellular costimulatory domains, or the clinical trial, MMLV-based CAR, denoted in as NTD, BBz, 28z, and MMLV-CD4z, respectively. Three weeks post injection, engraftment was measured to determine **(A)** baseline peripheral CD4 T cell counts and **(C)** baseline CAR^+^ CD8 T cell counts. Two days later mice were infected with HIV-1 Bal via tail vein injection. 22 days post infection, **(B)** endpoint peripheral CD4 T cell counts and **(D)** CAR^+^ CD8 T cell counts were obtained. **(E)** Seven and **(F)** eighteen days post infection mice were bled and HIV RNA copies per μl plasma were determined by qPCR and normalized to CD4 T cell counts. The non-normalized viral loads are displayed in S8 Fig. Mann Whitney Test was used to determine statistical significance (p values: ns >0.05, *<0.05, **<0.01, ***<0.0001).

CD19-specific CARs containing the CD28 signaling domain had superior *in vitro* activity than those containing the 4-1BB signaling domain, but the 4-1BB containing CARs proved superior in humanized mouse models and in patients [15, 23, 52]. We wished to determine whether the same was true for HIV-targeting CARs. In addition, we wanted to determine if our optimized CD4 CARs could control HIV better *in vivo* then the original CD4-zeta CAR that was tested in the clinic. To do this, we utilized a NSG humanized T cell (NSG hu-T cell) mouse model. In this model, detectable T cell engraftment (>10 cells per μl of blood) takes 2–3 weeks, and over the next 2–3 months T cell engraftment slowly rises until the mice become sick due to xenograft-mediated GVHD [53, 54]. HIV infection prevents CD4 T cell expansion and, paradoxically, makes the animals healthier. Thus, evidence of GVHD and high levels of CD4 T cell engraftment are strong evidence that anti-viral agents, such as CAR T cells in this case, are effective. Four groups of mice were compared with different CD8 effector cell populations: nontransduced (NTD), transduced with the optimized lentiviral vector containing either 4-1BB (BBz) or CD28 (28z) costimulatory domains, or transduced with the clinical trial MMLV-based vector (MMLV-CD4z). Pre-infection, baseline CD4 T cell counts did not differ significantly between the NTD, BBz, or 28z groups, and were significantly higher for the MMLV-CD4z treated mice (Fig 7A). After HIV infection, we observed that mice infused with T cells expressing the BBz or 28z construct had a 17-fold and 177-fold expansion of the number of human CD4 T cells, respectively (Fig 7B). In contrast, endpoint CD4 counts were depleted in NTD or MMLV-CD4z mice (Fig 7B). Examination of the number of CAR^+^ CD8 T cells in the different mouse cohorts revealed 389-fold, 587-fold, and 2-fold expansions in the BBz, 28z, and MMLV-CD4z T cells, respectively (Fig 7C and 7D), suggesting there is a correlation between the ability of CAR T cells to expand and the ability to protect CD4 T cells from HIV-mediated destruction.

We also examined viral loads. Since HIV replication is highly dependent on the number of CD4 T cells present in this model, we normalize viral load to the number of CD4 T cells present at a given timepoint to fairly compare different treatment groups. Seven days following HIV infection, BBz CAR T cells exhibited the greatest control over virus replication, with many mice showing undetectable virus loads, whereas plasma from NTD, 28z, and MMLV-CD4z treated animals contained approximately 1 normalized copy of HIV RNA per μl (Fig 7E). Eighteen days post infection, the median copy number of HIV RNA was reduced by more than 10-fold in both BBz and 28z treatment groups, compared to the mice that were treated with NTD T cells (Fig 7F). However, MMLV-CD4z treated mice had similar HIV RNA loads as NTD treated mice. Thus, the optimized CARs are superior at protecting CD4 T cells, promoting CD8 T cell expansion, and controlling HIV replication *in vivo*, with BBz CARs superior in preventing the early spread of HIV.

### CAR T cells containing 4-1BB outperform CAR T cells containing CD28 in an HIV-treatment model

After establishing that optimized CARs can function in humanized mice to control HIV replication, we next wanted to model CAR treatment of pre-established HIV infections and further examine whether 4-1BB or CD28 costimulation promoted better *in vivo* control. To mimic how CARs would be applied in a clinical trial, we injected CAR T cells into NSG mice with a previously established pool of HIV-infected T cells in the presence of ART and monitored virus rebound after ART was stopped (S9A Fig).

**Fig 8:**
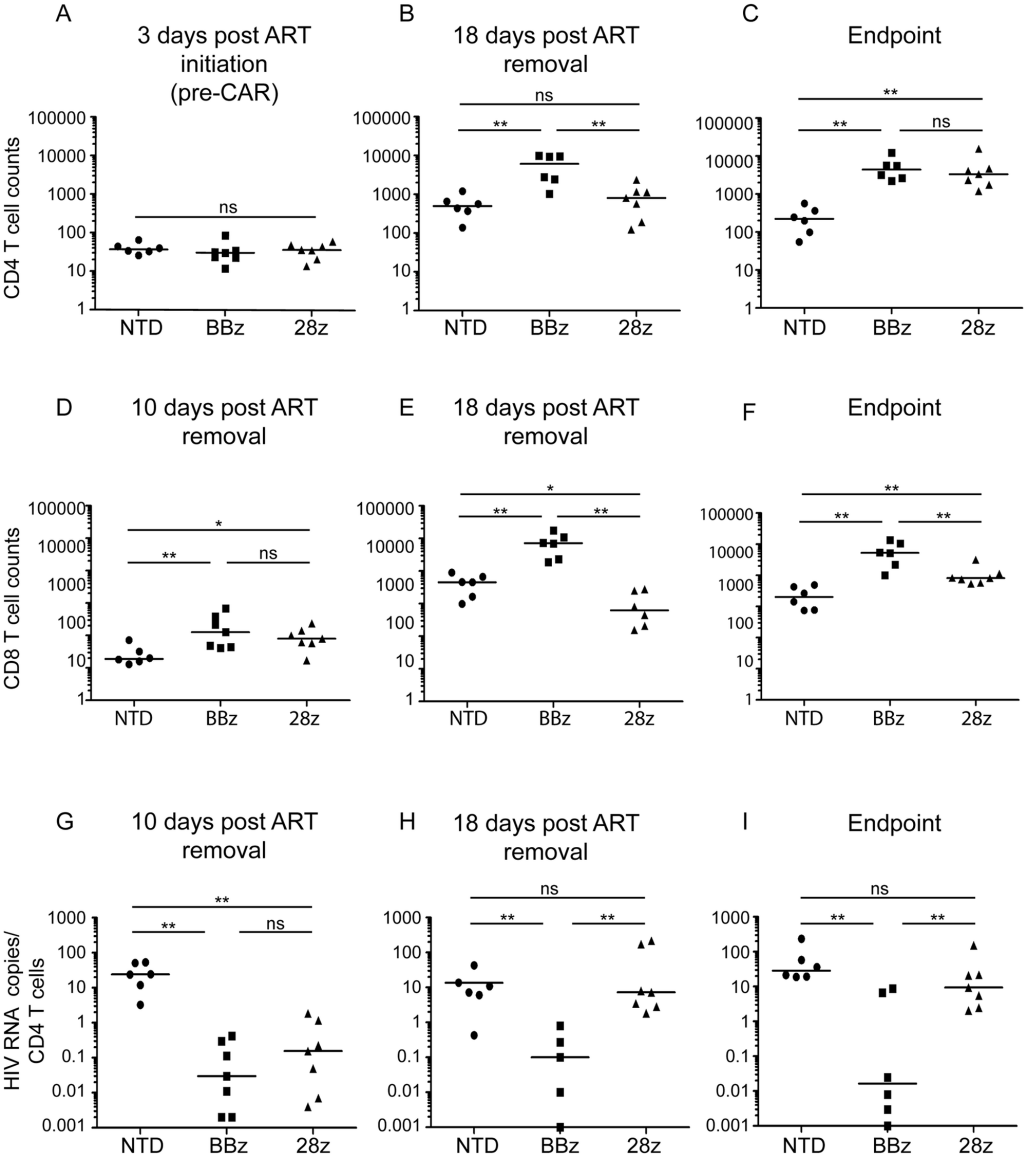
CAR T cells containing 4-1BB outperform CAR T cells containing CD28 in a humanized mouse HIV-treatment model. The experimental timeline and detailed description is provided in S8 Fig. CD4 T cell counts per μl of blood are shown (**A**) 3 days post ART initiation, prior to CD8 T cell injection (**B**) 18 days post ART removal and (**C**) at the endpoint termination bleeds (21 or 24 days post ART removal). For logistical reasons the mice had to be terminated in two groups, with BBz mice terminated 21 days post ART removal and the NTD and 28z terminated 24 days post ART removal. CD8 T cell counts are shown (**D**) 10 days post ART removal and CD8 T cell injection (**E**) 18 days post ART removal and (**F**) at the endpoint termination bleeds (21 or 24 days post ART removal). HIV RNA copies per μl plasma were determined by qPCR and normalized to CD4 T cell counts (**G**) 10 days post ART removal (**H**) 18 days post ART removal and (**I**) the endpoint bleed. The non-normalized viral loads are displayed in S12 Fig. Mann Whitney Test was used to determine statistical significance (p values: ns >0.05, *<0.05, **<0.01, ***<0.0001).

After three days of HIV infection in the presence of ART, the peripheral blood CD4 T cell counts were similar for all groups (Fig 8A). However, at 18 days post ART removal CD4 T cell depletion was apparent in NTD and 28z CAR treated mice, with significantly higher CD4 T cell counts in the BBz treatment group (Fig 8B). By the endpoint bleed, the 28z CARs demonstrated increased protection of CD4 T cells, and only the NTD mice had significantly lower CD4 T cell counts (Fig 8C). In contrast, mock infected mice maintained similar CD4 T cell counts in all treatment groups at all timepoints (S10A–S10C Fig). Interestingly, endpoint CD4 T cell counts were similar in this experiment for both BBz and 28z CAR treatment groups (Fig 8B and 8C), as opposed to Fig 7 where CD4 counts were significantly higher in 28z treated mice. Ten days post ART removal, the CAR treated mice had higher peripheral blood CD8 T cell counts compared to NTD mice (Fig 8D). This effect was HIV-specific, as all mock treated mice had similar CD8 T cell counts (S10D Fig). However, by 18 days post ART removal the CD8 T cell counts were significantly higher in mice that received BBz CARs compared to NTD or 28z CAR-treated mice (Fig 8E and 8F), and this was also seen in mock treated mice (S10E and S10F Fig), consistent with the notion that 4-1BB signaling promotes T cell persistence in the absence of antigen [23, 26, 55].

Prior to CD8 T cell injection, while the animals were on ART, most mice had undetectable viremia and 3/20 had very low levels of plasma HIV RNA (<1 copy per ul, S1 Table). Ten days post ART removal, all NTD mice had detectable plasma RNA, whereas all CAR treated mice had very low or undetectable HIV, as measured by plasma HIV RNA (Fig 8G). Similar patterns of control were detected via measuring plasma HIV p24 Gag protein using an ultrasentive assay (S11 Fig). However, after 18 days post ART removal, 28z treated mice experienced an increase in HIV replication and had similar plasma levels of HIV as NTD mice, whereas the BBz mice maintained significantly better control (Fig 8H and 8I and S11B Fig). Together, these data suggest that a CD4 CAR containing the 4-1BB zeta signaling domain will be most effective in HIV cure strategies because 1) its ability to act rapidly to prevent HIV spread (Fig 7E and 7F), 2) its ability to durably prevent viral rebound (Fig 8H and 8I), and 3) its ability to promote T cell survival in the absence of antigen (S10E and S10F Fig).

## Discussion

T cell control over virus replication is enabled through potent effector mechanisms that ensure rapid killing and prevent dissemination of progeny viruses [56]. However, HIV employs multiple strategies to evade T cell recognition and control. For instance, the HIV Nef protein modulates expression of MHC class I, CD28, and other proteins involved in immune recognition to evade cytotoxic T lymphocytes (CTLs) [3, 57]. Additionally, the phenomenal capacity of HIV to modify its MHC class I restricted peptide antigens promotes escape from CTL responses [4]. Moreover, due to chronic HIV persistence, CTLs become exhausted and progressively lose their effector functions [7]. For these reasons, there is a strong rationale to develop HIV-specific T cells with enhanced, supraphysiologic ability to control HIV replication for therapeutic studies aimed to establish long-term control, or a “functional cure,” in the absence of antiretroviral treatment.

We hypothesized that we could re-engineer the original CD4 CAR that was tested in the clinic and determined to be safe and long lived, but lacked potent antiviral activity, to develop T cells that were far more effective in controlling HIV infection [16–18]. We found that switching from a MMLV-based gammaretroviral vector to an HIV-based lentiviral vector resulted in much higher CAR surface expression, and expression was further augmented upon substituting the EF1α promoter for than the PGK promoter, consistent with previous cancer-targeting CAR studies [23]. Higher CD4 CAR expression in primary human CD8 T cells correlated with improved control over HIV replication. However, this was not a perfect correlation, as incorporating the CD8α TM domain rather than CD4 TM domain resulted in lower CAR expression but improved control over HIV replication. We favor two non-mutually exclusive explanations of this finding: the CD8α TM domain facilitates less HIV fusion than the CD4 TM domain, making cells less susceptible to CD4 CAR-mediated infection; and the CD8α TM domain promotes dimerization, which may potentiate signaling [23, 37, 39]. Indeed, improved control over HIV replication by CD4 CARs inversely correlated with the susceptibility of CAR^+^ CD8 T cells to become infected, suggesting that HIV infection limits CAR effector activity.

We found that, despite similar levels of specific lysis and stronger cytokine production in response to Env^+^ K562 cells, scFv-based CARs could not control HIV replication as well as CD4-based CARs *in vitro*, suggesting that CD4 may recognize HIV Env expressed on the cell surface faster than the scFvs we examined. Although it is certainly possible that additional and/or better optimized scFv CARs could have superior activity than those described here, we favor the use of T cells expressing the CD4 CAR due to its extensive clinical safety profile, lack of immunogenicity, and the dependence HIV shows for using CD4 as an entry factor [22]. While escape from antibody targeting is common in HIV, the reliance on CD4-mediated entry suggests that escape from CD4 binding will impose significant, if not lethal, fitness costs. As safety and efficacy of this re-engineered CAR are demonstrated in HIV-infected individuals, combinatorial approaches with scFv based CARs may further augment control of HIV replication in the absence of ART.

The improved control exhibited by the re-engineered CD4 CAR is impressive, with at least a 50-fold augmentation in control over HIV replication (S13 Fig). Many studies have used similar methods to study the ability of previous CD4 CAR designs and HIV-specific T cells to limit HIV replication *in vitro* and an E:T ratio of 1:1 was generally required to obtain complete control, in line with what we observed with these constructs [19–21]. The superiority of CARs compared to a TCR-based approach may be due to antigen-binding affinity, target cell-binding avidity, T cell activation kinetics, or bypassing the detrimental effects HIV Nef [3]. Overall, the potent control achieved by the re-engineered CARs provides optimism for clinical utility and achieving a functional cure.

We opted to use a NSG hu-T cell mouse model in which human T cells isolated from human donors would be manufactured in an analogous manner as a clinical trial. While there are certainly drawbacks to this model, such as GVHD and the inability to replace CD4 T cells once they are depleted by HIV infection, this model has been successfully used in preclinical, FDA mandated biotoxicity and efficacy studies [54, 58] and has mirrored the outcome of several clinical trials exploring gene therapy approaches to treat HIV infection [53, 54]. Our *in vivo* results demonstrated that the re-engineered CD4 CARs had potent antiviral activity, vastly superior to the original CD4-zeta CAR construct. Interestingly, we saw superior control by the 4-1BB containing CARs both early in infection within the HIV prevention model and more durable control at late timepoints within the treatment model, compared to mice treated with CD28 containing CARs. In contrast to the mouse data, our *in vitro* studies show that CD28 costimulation promoted higher cytokine production and better control over HIV replication, relative to a CD4 CAR containing 4-1BB costimulation.

One way to reconcile the difference between our *in vitro* and *in vivo* finding is to consider CAR T cell expansion kinetics. We showed *in vitro* that if the CAR^+^ CD8 T cells are in sufficient numbers, they can prevent the spread of infection in all cells; however, if they fall below a critical level, then both the CD4 T cells as well as the CAR^+^ CD8 T cells are infected and HIV begins to spread. In our HIV treatment model, effective early control results in an expansion of more CD4 T cell targets. If the CAR^+^ CD8 T cells fail to expand in proportion to the CD4 T cells or are depleted by HIV, then they may fall below the critical threshold required to control HIV replication. Differences in the relative expansions of BBz and 28z CARs *in vivo*, which may partially be due to better antigen-independent expansion of BBz, may underlie the differences in HIV control observed between Figs 5 and 8. In support of this hypothesis, we observed less 28z CD8 T cell expansion relative to BBz and this correlated to less viral control (Fig 8). Moreover, by the end of the HIV-prevention model, shown in Fig 7, similar control by either CAR resulted when the 28z CARs expanded to a similar degree as the BBz CARs.

In any case, one would predict that using CCR5 ZFNs [59], C34 based fusion inhibitors [58], or other methods to protect CD4 CAR expressing cells from HIV infection would further potentiate the therapeutic potential of CD4 CAR T cells. In fact, a recent paper using scFv based CARs demonstrated this by inserting the CAR into the CCR5 locus [60]. In addition, the ability to persist in the absence of antigen may be important for a functional cure, in which durable T cell control may need to last for decades. Together, our data suggests that CD4 CARs containing the 4-1BB costimulation domain may be the most effective way to deliver T cell control of HIV replication. Unfortunately, there is no animal model that faithfully mirrors HIV infection in humans, and issues of trafficking, immune privilege, and durability are difficult to fully model in humanized mice. Nonetheless, these data provide the rationale to re-visit the clinical utility of CD4 CAR in HIV-infected individuals and provide optimism for CAR T cells to achieving durable control over HIV in the in the absence of ART.

## Methods

### Plasmid construction

pRT43.2 GFP, the backbone of the original clinical trial vector, was obtained courtesy of Dr. Maribeth Eiden [19, 61] A restriction site linker was inserted into the PstI and SalI sites, removing the CMV promoter. The PGK promoter CD4-zeta sequence was amplified from pRRL.PGK.F3 (a gift of Dr. Tom Dull) with oligos 5’ GTATCGATCACGAGACTAGC and 5’TTAAACCGGTGTCTGGCCTTTGAGTGGTGA and inserted into XhoI and AgeI sites in the linker within pRT43.2. pTRPE CD4 zeta was created by amplifying the CD4 extracellular domain was amplified from pRRL.PGK.F3 with primers: 5' TTAATGGGATCCATGAACCGGGGAGTCCCTTT and 5' AAGGACTTCCGGATGGCTGCACCGGGGTGGACCATG-3' and inserted into the BamHI and BspE1 sites in the pTRPE backbone containing the CD8α extracellular hinge and transmembrane domains and the 4-1BB and CD3 zeta intracellular costimulatory domains [62]. pTRPE lentiviral vectors containing the CD8α hinge-CD8αTM-CD3ζ or the CD8α hinge-CD28TM-CD28-CD3ζ ICD were used as template to PCR amplify the hinge-TM-and ICD region into the BspE1 and Sal1 sites with primers:5’ GGGACACTCCGGAACCACGACGCCAGCGCCGCG and 5’ GGGACACGTCGACTTAGCGAGGGGGCA. A lentiviral vector that expressed a B57 restricted TCR capable of recognizing HIV p24Gag epitope KAFSPEVIPMF (pTRPE B57-KF11) was generated by synthesizing the TCRα and TCRβ gene sequence (IDT, the TCR sequences were a generous gift of Xu Yu and Bruce Walker [63]). The TCRα and TCRβ gene sequence was separated by the T2A for expression of both TCR genes as previously described [64]. VRC01, 3BNC60, PGT128, and PGDM1400 scFv CARs were generated from the published parental antibody sequences, with a light-linker-heavy chain configuration [46, 65–68]. The linker sequence is: GGSSRSSSSGGGGSGGGG. Amino acid sequences were codon-optimized (Geneart) and synthesized as double-stranded DNA fragments (IDT or Geneart), flanked with suitable restriction sites and cloned into pTRPE plasmids with the BamHI and BspE1 sites. The PG9 scFv was obtained as a generous gift from Dr. Phil Johnson and cloned into the pTRPE plasmid with the BamHI and BspE1 sites. The amino acid sequences are found in S18 Fig.

### Virus production and transduction

To generate lentiviral particles, expression vectors encoding VSV glycoprotein, HIV Gag and Pol, and Rev (pTRP pVSV-G, pTRP g/p.RRE pTRP.REV) were synthesized by DNA 2.0 and transfected onto HEK293T cells with pTRPE transfer vectors using the Lipofectamine 2000 transfection reagent (Invitrogen, Life Technologies) as previously described [69]. Transfected HEK293T supernatant was collected at 24- and 48-hour timepoints, filtered through 0.45 um nylon syringe filters, and concentrated by ultracentrifugation at 18 hours at 8,500RPM at 4°C. Medium was aspirated and pellet was resuspended in 1.2ml total volume and stored at -80°C. Murine retrovirus: 10^7^ 293T cells were plated and after 18 hours co-transfected with 20 ug pNGVL3-g/p, 20 ug pMSCV-RD114, and 40 ug pMMTV CD4 zeta transfer vector also using the Lipofectamine 2000 transfection reagent (Invitrogen, Life Technologies). After 24, 48, and 72 hours, supernatants were harvested, filtered through 0.45 um nylon syringe filters, and frozen at -80C.

### Cell culture

T cells were purified from normal donors by negative selection using the RosetteSep Human CD4+ or CD8+ T Cell Enrichment Cocktails according to the manufacturer’s protocols (StemCell Technologies). T cells were cultured at 1x10^6^ per mL in “complete RPMI 1640:” RPMI 1640 (Life Technologies) supplemented (ThermoFisher Scientific) wih 10% fetal calf serum (Seradigm), 1% Penn Strep (Life Technologies), 2 mM GlutaMax (Life Technologies), and 25 mM HEPES buffer (Life Technologies). T cells were stimulated with anti-CD3/CD28 coated Dynabeads (Life Technologies) at a 3:1 bead to cell ratio and 100–300 IU/mL of recombinant human interleukin-2 for 5 days prior to bead removal. 1 day after stimulation, 200ul of lentivirus supernatant was added to 0.5x10^6^ cells so that between 40–70% of the T cells would be transduced. MMLV vector transduction was performed on days 3 and 5, with 1ml virus supernatant added to a Retronectin (Takara)-coated 24 well plate and spinoculated according to the manufacturer’s instructions. Medium was doubled on day 3 and changed completely on day 5, and then added every other day throughout cell culture, or as necessary based on cell counts.

### *In vitro* HIV replication control assay and intracellular Gag stain

Two days after removing the anti-CD3/CD28 beads, CD4 T cells were infected with the CCR5-tropic HIV strain Bal, and 24 hours later were co-cultured at varying effector to target (E:T) ratios with CAR CD8 T cells. Bal viral stocks (280ng/ml p24) was prepared by harvesting the cell-free supernatant from anti CD3/CD28 activated CD4 T cells and freezing in aliquots. Activated CD4 T cells were infected by adding approximately 1ml of supernatant per 20 million cells 2–3 days after removing beads. The following day CD4 and CD8 T cells were co-cultured at varying E:T ratios and HIV spread was monitored by intracellular p24 Gag with the KC57 anti-Gag-RD1 antibody (Beckman Coulter) and the Invitrogen Fix and Perm buffers, according the manufacturers’ instructions, gating on a population of uninfected cells. To ensure that the same numbers of CAR^+^ CD8 T cells were being compared, we diluted out populations with higher transduction efficiencies by adding in nontransduced T cells until all CAR^+^ CD8 T cell populations matched the population with the lowest CAR or TCR transduction efficiency.

### Flow cytometry

CD4 CAR surface expression was monitored with mouse anti human CD8-FITC and anti-human CD4 APC antibodies (BD biosciences). The scFv CARs were detected with biotinylated F(ab')2 goat anti-human IgG (Jackson) and Streptavidin-PE (BD biosciences). HLA-DR was detected on Raji B cells and K562 cells originally obtained from the American Type Culture Collection (ATCC) using a mouse anti-human HLA-DR PE antibody (BD biosciences). Cells were visualized on a LSR II flow cytometer (BD Biosciences) and analyzed using Flowjo software (Tree Star) as previously described [70]. B57-KF11 TCR transduction efficiency was detected with an antibody to the TCR Vβ17 chain, subtracting the background Vβ17 signal from the NTD T cells (S5A Fig).

### *In vitro* cytotoxicity and cytokine assays

*In vitro* killing of K562 cell derived targets was tested with a ^51^Cr-release assay. 5x10^5^ target cells were loaded with 50 mCi of Na_2_^51^CrO_4_ (Perkin Elmer) for 90–120 minutes, washed twice and resuspended in phenol red-free medium with 5% FBS. NTD, CD4 CAR, or scFv CAR transduced T cells (two weeks after initial activation) were co-incubated with loaded YU2 Env^+^ K562 target cells for 4 hours at various E:T ratios, and chromium release into the supernatant was measured with a MicroBeta2 plate counter (Perkin Elmer). Intracellular cytokine production was measured after co-culturing 5x10^5^ NTD, CD4 CAR, or scFv CAR transduced CD8 T cells at a 1:1 E:T ratio with the various target K562 cell populations for 6 hours. Cytokine production was detected as previously described [71] using rat anti human IL-2 APC (BD biosciences), mouse anti human MIP-1β PerCP Cy5.5 (BD biosciences), mouse anti human IFN-γ FITC (BD biosciences), and mouse anti human CD107a PE (BD biosciences), along with Invitrogen Fix and Perm buffers.

### Vector integration qPCR

Genomic DNA was isolated from transduced CD8 T cells with the iPrep Purification Instrument (Thermo fisher scientific) and qPCR analysis was performed using ABI Taqman technology, with a modified version of the previously described assay designed to detect the integrated CD4-zeta sequence in genomic DNA (gDNA) [22]. To determine copy number per unit DNA, a standard curve was generated consisting of 5 to 10^6^ plasmid copies spiked into 200 ng nontransduced control gDNA. The plasmid copy number in the standard curve was verified using digital qPCR with the same CD4-z primer/probe set, and performed on a QuantStudio 3D digital PCR instrument (Life Technologies). Each data-point was evaluated in triplicate with a positive Ct value and % CV less than 0.95% for all quantifiable values. To control for the quantity of interrogated DNA, a parallel amplification reaction was performed using 10 ng gDNA and a primer/probe set specific for a non-transcribed genomic sequence upstream of the CDKN1A (p21) gene as previously described [72]. These amplification reactions generated a correction factor to adjust for calculated versus actual DNA input. Copies of transgene per cell were calculated according to the formula: [Average copies of transgene(from qPCR)x gDNA input Correction Factor/Input gDNA(ng)]x 0.0063 ng gDNA/cell.

### HIV prevention humanized mouse model

6 week old NSG (NOD-*scid* IL2Rg^null^) mice were obtained from The Jackson Laboratory (JAX) and at 7 weeks treated with 30mg/kg Busulfan mixed 1:1 with PBS. 24 hours later mice were injected via tail vein with 10x10^6^ human lymphocytes in 100ul 0.5% human serum albumin in PBS, comprised of 8 million CD4 T cells and 2 million CD8 T cells (NTD, BBz, 28z, or MMLV-CD4z transduced with a 50% transduction efficiency). Three weeks later mice were tail vein injected with 15ng HIV Bal mixed 1:1 with PBS. Peripheral blood was obtained by retro-orbital bleeding, and human CD4 and CAR^+^ CD8 lymphocyte counts were enumerated using BD lysis buffer and BD TruCount tubes as previously described [73], staining with mouse anti human CD45 PerCp Cy5.5 (BD Biosciences), mouse anti human CD4 BV421 (Biolegend), and mouse anti human CD8α BV711 (Biolegend).

### HIV treatment humanized mouse model

5 week old NSG (NOD-*scid* IL2Rg^null^) mice were obtained from The Jackson Laboratory (JAX) and at 6 weeks were injected with 5 million CD8-depleted human PBMCs, and 12 days later injected with 1 million HIV Bal-infected (or mock-infected) autologous CD4 T cells that had been *in vitro* infected with HIV Bal and cultured with ART for 2 days prior to freezing. The same day as HIV infection, mice began receiving 200mg/kg daily intraperitoneal injections of the reverse transcriptase inhibitor nucleotide analog tenofovir disoproxil fumarate (TDF) for 4 days. On Day 16, 5 million CD8 T cells were injected (NTD, BBz, or 28z). Peripheral blood was obtained by retro-orbital bleeding, and human CD4 and CAR^+^ CD8 lymphocyte counts were enumerated using BD lysis buffer and BD TruCount tubes as previously described [73], staining with mouse anti human CD45 PerCp Cy5.5 (BD Biosciences), mouse anti human CD4 BV421 (Biolegend), and mouse anti human CD8α BV711 (Biolegend).

### HIV RNA viral load assay

RNA was extracted from 10–30μl of plasma using methods as described [74] and reconstituted in a final volume of 15ul. Prior to extraction, a uniform quantity of Replication Competent Avian Sarcoma (RCAS) virus spiked into each plasma sample and amplified separately to verify virus/RNA recovery and absence of PCR inhibition [75]. RNA was reverse transcribed using random hexamers and quantified by Q-PCR using the LightCycler 480 Probes Master (Roche; Indianapolis, IN) on an ABI 7500FAST real-time thermocycler using an *in vitro* transcribed RNA standard. For each sample, the Q-RT-PCR reaction was run in duplicate on 5ul RNA; no-reverse transcriptase reaction and RCAS amplification were run on one well per sample using 2.5ul RNA. The HIV-1 primer/probe targets the *pol* gene and detects all group M clades as described in [74], and RCAS amplification used primer/probe as described in [75]. HIV-1 quantification was normalized to equivalent volumes of starting plasma. Where indicated, viral load was expressed as number of RNA copies per CD4 T cell.

### Culture supernatant p24 detection

Culture supernatant was harvested after 7 days of co-culture and diluted 1:10,000 and analyzed using the commercially available p24 ELISA assay kit (Perkin-Elmer). Assay protein standards ranged from 9.4pg/ml to 150pg/ml.

### Ultrasensitive p24 detection from mouse plasma

Plasma was collected by centrifugation of the whole blood and diluted according to a protocol supplied by Bonnie Howell (Merck & Co, Inc.). The HIV p24 Gag protein was measured using the p24 single molecule array using the Simoa HD-1 Analyzer (Quanterix) following the manufacturer’s instructions. Each sample was measured in duplicate and concentration calculated based on a standard curve. The average concentration of two replicates for each sample was reported. The accurate detection range was 0.008pg/ml to 39.5pg/ml.

### Statistics

*In vitro* HIV replication control significance was detected using a 1-way ANOVA test, stratifying based on the E:T ratio (p values: ns >0.05, *<0.05, **<0.01, ***<0.0001), using the 30:1 E:T ratio for Fig 4A. All E:T ratios are presented in the figures as a single graph due to space limits. For the mouse models, non-parametric distributions were determined and Kruskall Wallis analysis was performed and, if overall comparison showed significant differences, then Mann Whitney Test was performed for pairwise comparisons (as samples were not powered for post-hoc analysis of multiple comparisons) and significance results are reported on each figure (p values: ns >0.05, *<0.05, **<0.01, ***<0.0001).

### Ethics statement

Purified CD4 and CD8 T lymphocytes were obtained by University of Pennsylvania Human Immunology Core/CFAR Immunology Core from de-identified healthy donors. All humanized mouse experiments were approved by the University of Pennsylvania’s Institutional Animal Care and Use Committee (Protocol 804563) and were carried out in accordance with recommendations in the Guide for the Care and Use of Laboratory Animals of the National Institutes of Health.

## Supporting information

S1 Fig Gating strategy to separate the CD8 T cells from the CD4 T cells. After setting up our coculture assay, described in the Fig 1 legend, HIV replication is measured by staining for intracellular p24 (Gag). To distinguish between HIV spread throughout the CD4 T cells and infection of the CD4 CAR^+^ CD8 T cells, separate gates are drawn on these two populations. After gating for **(A)** cell size (FSC versus SSC plot), **(B)** CD8 and CD4 are plotted and two gates are drawn: **(D)** one encompasses all CD8^+^ cells and will encompass CD8 single positive nontransduced cells or CD4^+^ CD8^+^ double positive, CAR transduced CD8 T cells. The other gate (**C**) is on CD8 negative cells, to capture infected cells that have downregulated CD4 as well as CD4 expressing cells.
(PNG)

S2 Fig CD4 CAR Transduced CD8 T cells are not infected by cell-free HIV. Primary human CD8 T cells were activated and either left NTD or transduced with an optimized CD4 CAR lentiviral expression vector (EF1α promoter, CD8α transmembrane domain). After eight days, the cells were either left uninfected, inoculated with 70ng p24 of HIV Bal by cell-free addition to culture supernatant, or cocultured at varying effector to target ratios with CD4 T cells that had been previously infected with the same stock of HIV Bal for 24 hours (20ng p24/1x10^6^ CD4 T cells). After 6 days of culture, cultures were collected, and the CD8 T cells were gated on and analyzed for intracellular HIV Gag expression.
(PNG)

S3 Fig Supernatant HIV Gag p24 ELISA results correlate with intracellular HIV Gag p24 staining and flow cytometry. Using the experimental design described in the Fig 1 legend, a coculture assay was performed with the indicated CAR^+^ CD8 T cell populations with HIV-infected CD4 T cells. After 7 days of culture, the intracellular p24 Gag was measured by flow cytometry and the culture supernatant from the same wells was analyzed for p24 Gag by ELISA. Error bars indicate SEM (n = 3).
(PNG)

S4 Fig Gag staining in CAR^+^ CD8 T cells is not an artifact of gating on a small number of CD8 T cells and CD4 CAR construct is not downregulated by HIV infection. Using the experimental design described in the Fig 1 legend, a coculture was performed using CD8 T cells either left NTD or transduced with an optimized CD4 CAR lentiviral expression vector (EF1α promoter, CD8α transmembrane domain). After 5 days of co-culture, the intracellular Gag was measured by flow cytometry, collecting 2 million cells per well to ensure that at the 1:200 dilution, 1x10^4^ CD8 T cells would be collected. The pattern of infection was compared to that seen in the same construct used in Fig 2 and presented as zebra plots. (**A**) Shows gating on CD8 positive cells and (**B**) shows gating on CD8 negative cells. **(C)**. CD8 T cells transduced with the optimized CD4 CAR containing 4-1BB costimulation were cultured at a 1:100 effector to target ratio with CD4 T cells infected with HIV Bal. At 3, 5 and 7 days of coculture, intracellular Gag was measured by flow cytometry to assess HIV infection and CD4 expression of CD8 negative cells and CD8 positive cells.
(PDF)

S5 Fig KF11 TCR-transduced CD8 T cells recognize Gag peptides presented by CD4 T cells. (**A**) Primary human CD8 T cells were obtained from a HLA-B57^+^ normal donor and activated with αCD3/αCD28 coated beads. Cells were either left nontransduced (NTD) or transduced to express a HLA-B57 restricted TCR specific for KAFSPEVIPMF (KF11). KF11 TCR transduction efficiency was detected with an antibody to the TCR Vβ17 chain, subtracting the background Vβ17 signal from the NTD T cells. (**B**) Primary human CD8 T cells from a HLA-B57^+^ T cell donor were activated with αCD3/αCD28 coated beads and were either left nontransduced (NTD) or transduced with a lentiviral vector expression vector for the KF11 TCR, frozen 8 days post activation, and then thawed 48 hours prior to coculture. Autologous CD4 T cells were activated with αCD3/αCD28 coated beads and 11 days post activation 10 million cells were electroporated with 40ug of mRNA encoding the HIV Gag or HIV Pol proteins, or mock electroporated. After 24 hours, the NTD or KF11 CD8s were cocultured in at a 1:3 E:T ratio for 5 hours and IL-2 and TNFα production was measured.
(PNG)

S6 Fig ScFv-based HIV specific CARs produce cytokines as well as CD4-based CAR do not control HIV replication as well as the CD4 CAR and succumb to infection. **(A)** Primary human CD8 T cells were activated either left NTD or transduced with the indicated CAR vectors. Two weeks post activation, the CD8 T cells were co-cultured for 6 hours at a 1:1 ratio with K562 cells expressing HIV-1 YU2 GP160, and intracellular IFNγ and MIP-1β production was measured. Transduction efficiencies were normalized to 60% prior to co-culture. **(B)** Using the experimental design summarized in Fig 1, the HIV-specific CARs were tested for their ability to control HIV-1 replication in primary human CD4 T cells. NTD, GFP transduced, and CD19-zeta CAR transduced CD8 T cell treatments were included as controls. After 6 days of co-culture, intracellular Gag and CD4 staining is shown for CD8 negative T cells. **(C)** Shows gating on the CD8 positive cells.
(PNG)

S7 Fig HLA-DR expression histogram. K562 cells transduced with vectors encoding the HLA-DR*0401 α and β chains and single-clone sorted on high expressing cells, were stained for HLA-DR expression along with K562 control cells that had been transduced with HLA-A2, and the MHC class II highly expressing Raji B cells.
(PNG)

S8 Fig Non-normalized HIV RNA viral loads for the HIV prevention mouse model. At **(A)** seven and **(B)** eighteen days post infection mice were bled and HIV RNA copies per μl plasma were determined by qPCR, corresponding to the graphs shown in Fig 7E and 7F, respectively. Mann Whitney Test was used to determine statistical significance (p values: ns >0.05, *<0.05, **<0.01, ***<0.0001).
(AI) Click here for additional data file.

S9 Fig HIV treatment model timeline. NSG (NOD-*scid* IL2Rg^null^) mice were injected with 5 million CD8-depleted human PBMCs, and 12 days later injected with 1 million HIV Bal-infected (or mock-infected) autologous CD4 T cells. For four days the infected mice were injected intraperitoneally with 200mg/kg of the reverse transcriptase inhibitor nucleotide analog tenofovir disoproxil fumarate (TDF) to prevent HIV viremia. Mice were bled on day 15 to analyze pre-CD8 human cell counts and measure plasma HIV RNA. On Day 16, 5 million CD8 T cells were injected that were either nontransduced or transduced with optimized CD4-zeta CARs containing either 4-1BB or CD28 intracellular costimulatory domains. CD8 T cell transduction efficiencies were normalized to 55% prior to injection into mice. Mice were bled on day 26 and 34. For logistical reasons the mice had to be terminated in two groups, with BBz mice terminated on day 37 and the NTD and 28z terminated on day 40.
(AI) Click here for additional data file.

S10 Fig CD4 and CD8 T cell counts for mock infected NSG mice used in the HIV treatment model. **(A-C)** CD4 T cell counts for the mock-infected (HIV-free) components of the mouse cohorts described in the Fig 8 legend and shown in Fig 7A–7C, (**A**) 3 days post mock infection, (**B**) 3 weeks post mock infection, and (**C**) endpoint. **(D-F**) CD8 T cell counts for the mock-infected (HIV-free) components of the mouse cohorts described in the Fig 8 legend and shown in Fig 7D–7F, (**D**) 2 weeks post mock infection, (**E**) 3 weeks post mock infection, and (**F**) endpoint. Mann Whitney Test was used to determine statistical significance (p values: ns >0.05, *<0.05, **<0.01, ***<0.0001). (**G**) Table of the median CD4 T cell counts at the different timepoints measured for both HIV-infected and mock infected groups.
(AI) Click here for additional data file.

S11 Fig Measures of HIV p24 Gag protein levels in plasma as detected by ultrasensitive p24 ELISA. Plasma was isolated from whole blood by centrifugation and diluted according to a protocol supplied by Bonnie Howell (Merck & Co, Inc.) and the HIV p24 Gag protein was measured p24 single molecule array using the Simoa HD-1 Analyzer. Values displayed are femtograms p24 per μl plasma normalized to CD4 T cell counts (**A**) 10 days post ART removal and (**B**) the endpoint bleeds, 21 or 24 days post ART removal. Mann Whitney Test was used to determine statistical significance (p values: ns >0.05, *<0.05, **<0.01, ***<0.0001).
(AI) Click here for additional data file.

S12 Fig Non-normalized HIV RNA viral loads for the HIV treatment mouse model. At **(A)**10 days post ART removal **(B)** 18 days post ART removal and **(C)** the endpoint bleed mice were bled and HIV RNA copies per μl plasma were determined by qPCR, corresponding to the graphs shown in Fig 8G–8I, respectively. Mann Whitney Test was used to determine statistical significance (p values: ns >0.05, *<0.05, **<0.01, ***<0.0001).
(AI) Click here for additional data file.

S13 Fig Summary of improvements made to original clinical trial vector. **(A)** Table and schematic depicting the complete list of modifications explored to improve the original clinical trial, MMLV-based construct. **(B)** Using the experimental design summarized in Fig 1E, primary human CD8 T cells were activated with αCD3/αCD28 coated beads and were either left nontransduced (NTD), transduced with the original MMLV-based CD4 based CAR driven by the PGK promoter (clinical trial CAR), or transduced the optimized EF1α-CD8α TM CAR, placed in a HIV-based lentiviral vector. Transduction efficiencies were normalized to 60% prior to co-culture. After 7 days of co-culture with HIV Bal-infected CD4 T cells, the expression of surface CD4 and intracellular Gag p24 was measured by flow cytometry, gating on CD8 negative T cells. **(C)** Shows gating on the CD8 positive cells. **(D)** Summary data for a single experiment performed in triplicate, gating on the CD8 negative cells. Error bars indicate SEM (n = 3). Significance was detected using a 1-way ANOVA test, stratifying based on the E:T ratio (p values: ns >0.05, *<0.05, **<0.01, ***<0.0001). This data is representative of three independent experiments.
(AI) Click here for additional data file.

S14 Fig Triplicate experiments demonstrating that a lentiviral backbone augments CD4 CAR expression and control over HIV replication. The same experimental setup (as described in the Fig 1 legend) was performed in triplicate and intracellular HIV Gag is shown at the peaks of HIV replication for three independent donors, gating on the CD8 negative cells. Error bars indicate SEM (n = 3). Significance was detected using a 1-way ANOVA test, stratifying based on the E:T ratio (p values: ns >0.05, *<0.05, **<0.01, ***<0.0001).
(AI) Click here for additional data file.

S15 Fig Triplicate experiments demonstrating that the EF1α promoter and CD8α transmembrane domains improve CAR expression and control over HIV-1. The same experimental setup (as described in the Fig 2 legend) was performed in triplicate and intracellular HIV Gag is shown at the peaks of HIV replication for three independent donors, gating on the CD8 negative cells. Error bars indicate SEM (n = 3). Significance was detected using a 1-way ANOVA test, stratifying based on the E:T ratio (p values: ns >0.05, *<0.05, **<0.01, ***<0.0001).
(AI) Click here for additional data file.

S16 Fig Triplicate experiments demonstrating that the CD4 CAR is over 100-fold more potent than HIV-specific elite controller TCR *in vitro*. The same experimental setup (as described in the Fig 3 legend) was performed in triplicate and intracellular HIV Gag is shown at the peaks of HIV replication for three independent donors, gating on the CD8 negative cells. Error bars indicate SEM (n = 3). Significance was detected using a 1-way ANOVA test, stratifying based on the E:T ratio (p values: ns >0.05, *<0.05, **<0.01, ***<0.0001).
(AI) Click here for additional data file.

S17 Fig Triplicate experiments demonstrating that the CD4 CAR controls HIV-1 more effectively than broadly neutralizing antibody based CARs. The same experimental setup (as described in the Fig 4 legend) was performed in triplicate and intracellular HIV Gag is shown at the peaks of HIV replication for three independent donors, gating on the CD8 negative cells. Error bars indicate SEM (n = 3). Significance was detected using a 1-way ANOVA test, stratifying based on the E:T ratio (p values: ns >0.05, *<0.05, **<0.01, ***<0.0001).
(AI) Click here for additional data file.

S18 Fig Triplicate experiments demonstrating that CD28 and 4-1BB costimulation have opposing effects on the control of HIV-1 replication *in vitro*. The same experimental setup (as described in the Fig 5 legend) was performed in triplicate and intracellular HIV Gag is shown at the peaks of HIV replication for three independent donors, gating on the CD8 negative cells. Error bars indicate SEM (n = 3). Significance was detected using a 1-way ANOVA test, stratifying based on the E:T ratio (p values: ns >0.05, *<0.05, **<0.01, ***<0.0001).
(AI) Click here for additional data file.

S19 Fig Annotated Sequence Files for Optimized CD4 CARs and antibody based CARs. (DOCX)

S1 Table Copies of HIV RNA per μl plasma normalized to CD4 T cell counts while on ART. Mice were injected with 1 million HIV-infected CD4 T cells (See S8 Fig for timeline) and given daily intraperitoneal injections (200mg/kg) of the reverse transcriptase inhibitor nucleotide analog tenofovir disoproxil fumarate (TDF) for 3 days and then bled for viral load detection. HIV RNA copies per μl plasma were determined by qPCR and normalized to CD4 T cell counts.
(PNG)
