# Supplementary figures and images for: Supraphysiologic control over HIV-1 replication mediated by CD8 T cells expressing a re-engineered CD4-based chimeric antigen receptor
Source: PLoS Pathog. 2017 Oct 12;13(10):e1006613. doi: 10.1371/journal.ppat.1006613 (PMC5638568; doi:10.1371/journal.ppat.1006613)

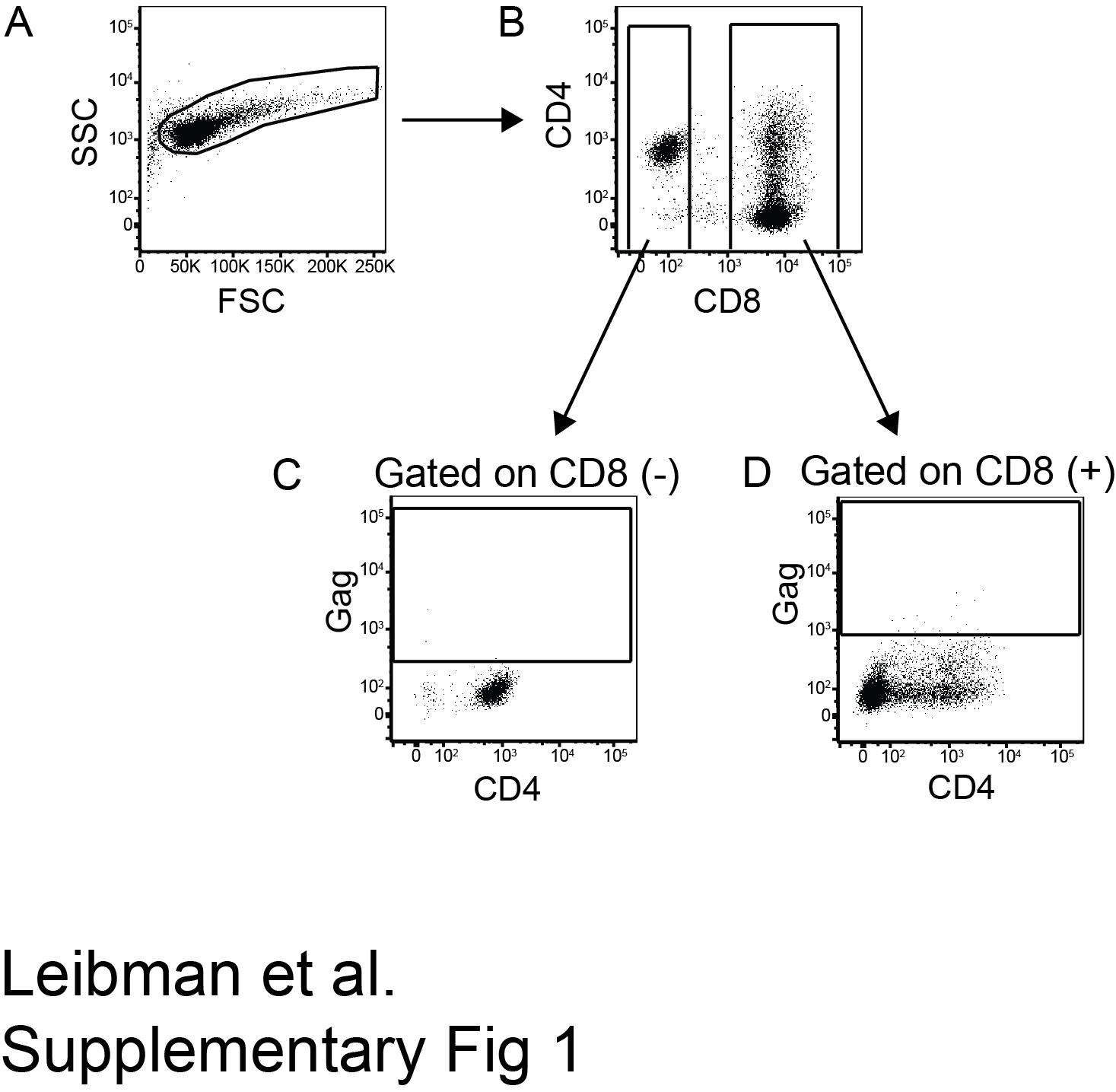

Supplement: S1 Fig — After setting up our coculture assay, described in the Fig 1 legend, HIV replication is measured by staining for intracellular p24 (Gag). To distinguish between HIV spread throughout the CD4 T cells and infection of the CD4 CAR+ CD8 T cells, separate gates are drawn on these two populations. After gating for (A) cell size (FSC versus SSC plot), (B) CD8 and CD4 are plotted and two gates are drawn: (D) one encompasses all CD8+ cells and will encompass CD8 single positive nontransduced cells or CD4+ CD8+ double positive, CAR transduced CD8 T cells. The other gate (C) is on CD8 negative cells, to capture infected cells that have downregulated CD4 as well as CD4 expressing cells. (PNG) [file ppat.1006613.s001.png]

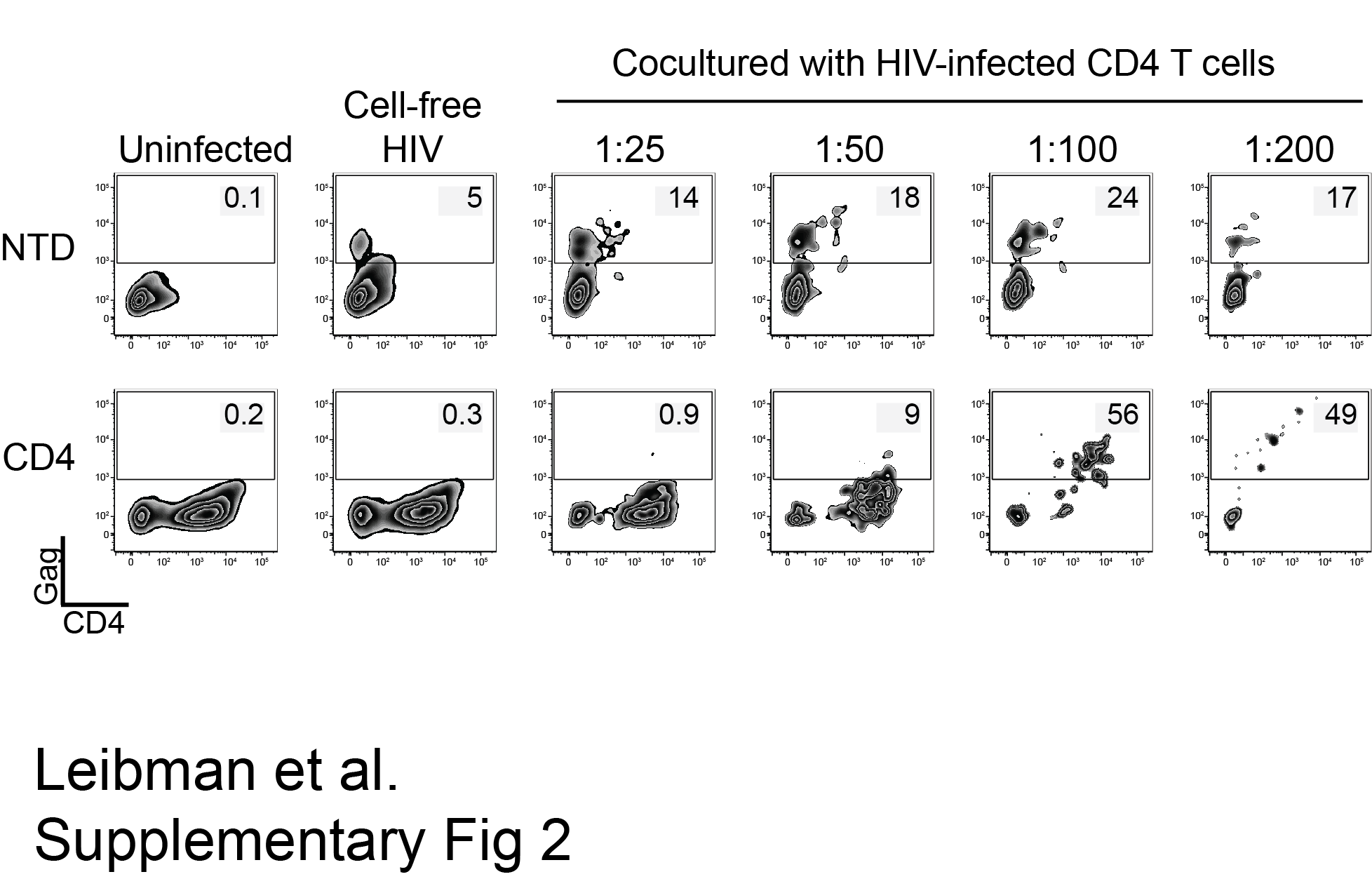

Supplement: S2 Fig — Primary human CD8 T cells were activated and either left NTD or transduced with an optimized CD4 CAR lentiviral expression vector (EF1α promoter, CD8α transmembrane domain). After eight days, the cells were either left uninfected, inoculated with 70ng p24 of HIV Bal by cell-free addition to culture supernatant, or cocultured at varying effector to target ratios with CD4 T cells that had been previously infected with the same stock of HIV Bal for 24 hours (20ng p24/1x106 CD4 T cells). After 6 days of culture, cultures were collected, and the CD8 T cells were gated on and analyzed for intracellular HIV Gag expression. (PNG) [file ppat.1006613.s002.png]

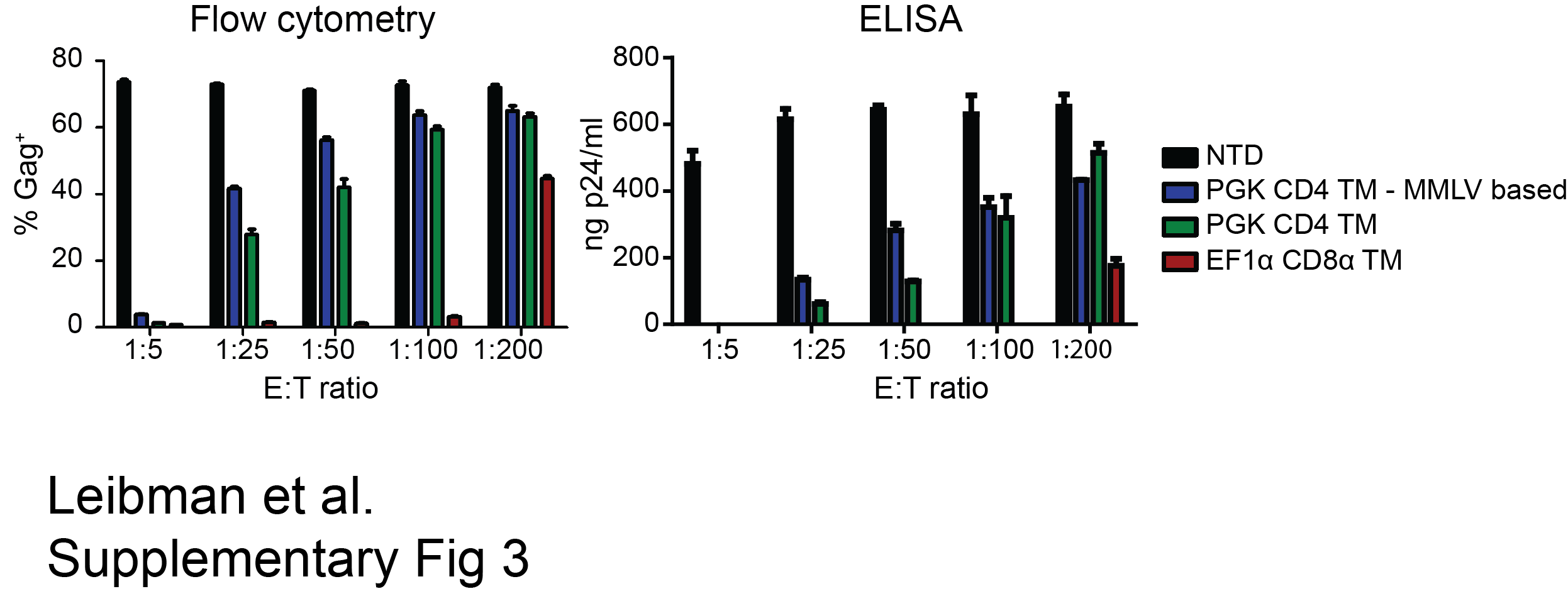

Supplement: S3 Fig — Using the experimental design described in the Fig 1 legend, a coculture assay was performed with the indicated CAR+ CD8 T cell populations with HIV-infected CD4 T cells. After 7 days of culture, the intracellular p24 Gag was measured by flow cytometry and the culture supernatant from the same wells was analyzed for p24 Gag by ELISA. Error bars indicate SEM (n = 3). (PNG) [file ppat.1006613.s003.png]

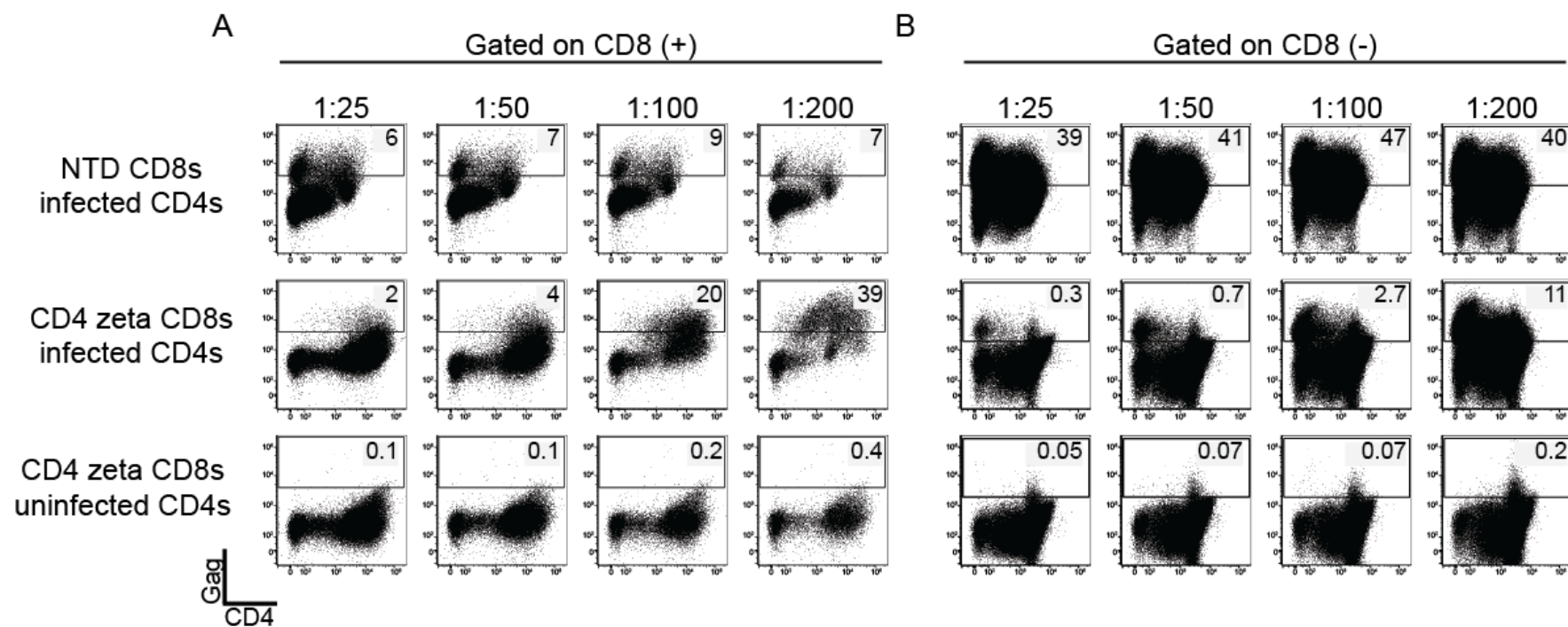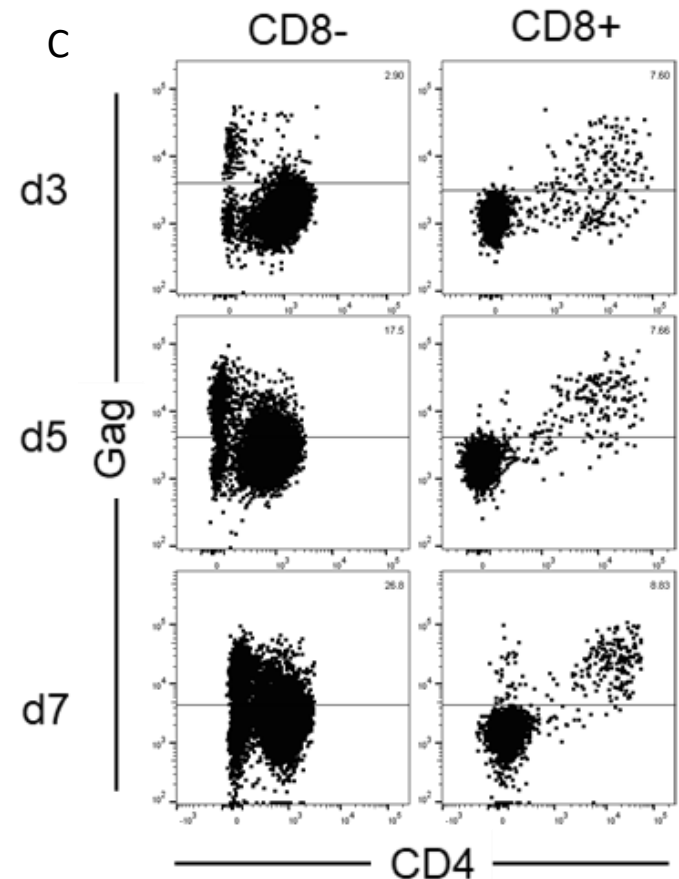

Supplement: S4 Fig — Using the experimental design described in the Fig 1 legend, a coculture was performed using CD8 T cells either left NTD or transduced with an optimized CD4 CAR lentiviral expression vector (EF1α promoter, CD8α transmembrane domain). After 5 days of co-culture, the intracellular Gag was measured by flow cytometry, collecting 2 million cells per well to ensure that at the 1:200 dilution, 1x10^4^ CD8 T cells would be collected. The pattern of infection was compared to that seen in the same construct used in Fig 2 and presented as zebra plots. (A) Shows gating on CD8 positive cells and (B) shows gating on CD8 negative cells. (C). CD8 T cells transduced with the optimized CD4 CAR containing 4-1BB costimulation were cultured at a 1:100 effector to target ratio with CD4 T cells infected with HIV Bal. At 3, 5 and 7 days of coculture, intracellular Gag was measured by flow cytometry to assess HIV infection and CD4 expression of CD8 negative cells and CD8 positive cells. (PDF) [file ppat.1006613.s004.pdf]

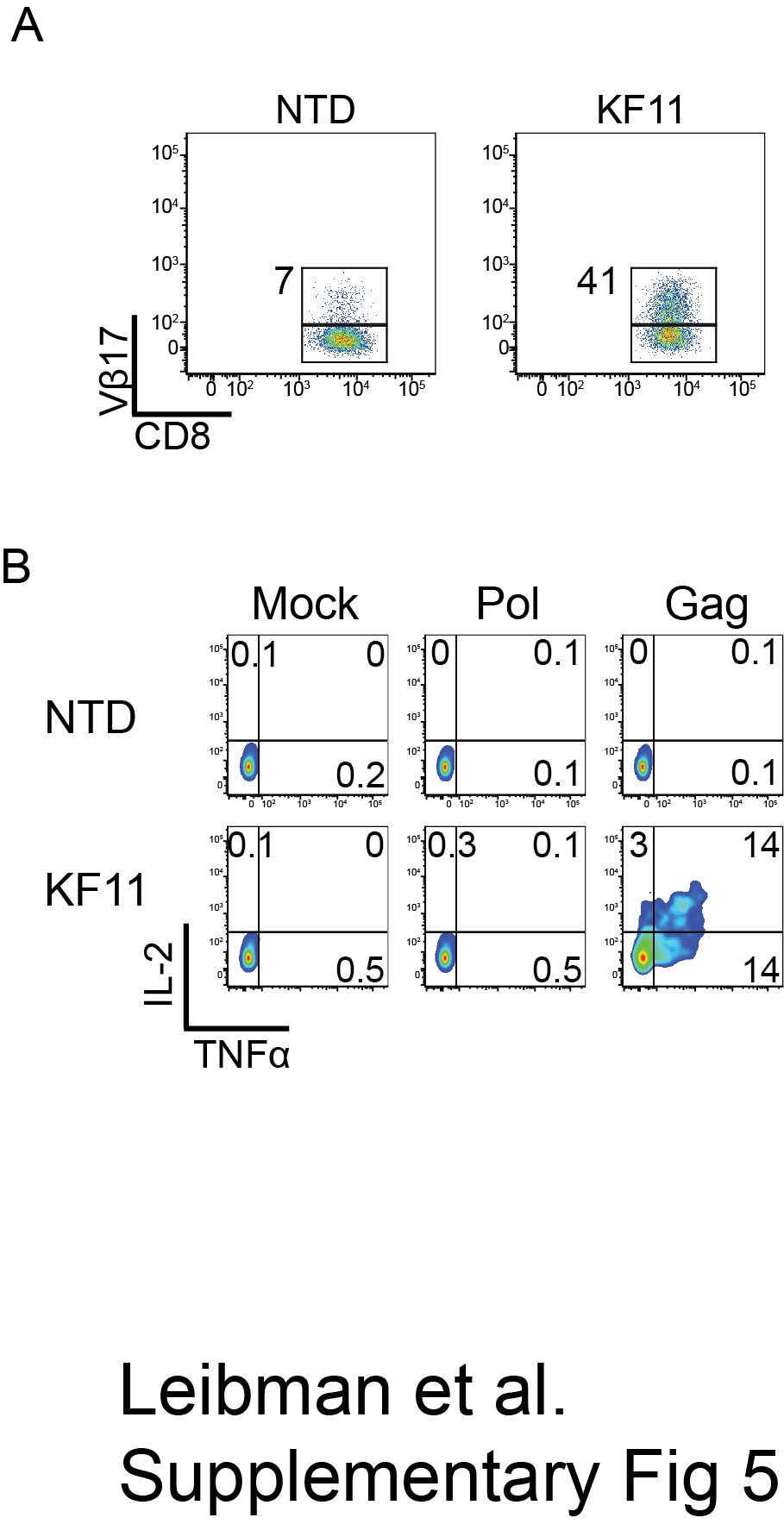

Supplement: S5 Fig — (A) Primary human CD8 T cells were obtained from a HLA-B57+ normal donor and activated with αCD3/αCD28 coated beads. Cells were either left nontransduced (NTD) or transduced to express a HLA-B57 restricted TCR specific for KAFSPEVIPMF (KF11). KF11 TCR transduction efficiency was detected with an antibody to the TCR Vβ17 chain, subtracting the background Vβ17 signal from the NTD T cells. (B) Primary human CD8 T cells from a HLA-B57+ T cell donor were activated with αCD3/αCD28 coated beads and were either left nontransduced (NTD) or transduced with a lentiviral vector expression vector for the KF11 TCR, frozen 8 days post activation, and then thawed 48 hours prior to coculture. Autologous CD4 T cells were activated with αCD3/αCD28 coated beads and 11 days post activation 10 million cells were electroporated with 40ug of mRNA encoding the HIV Gag or HIV Pol proteins, or mock electroporated. After 24 hours, the NTD or KF11 CD8s were cocultured in at a 1:3 E:T ratio for 5 hours and IL-2 and TNFα production was measured. (PNG) [file ppat.1006613.s005.png]

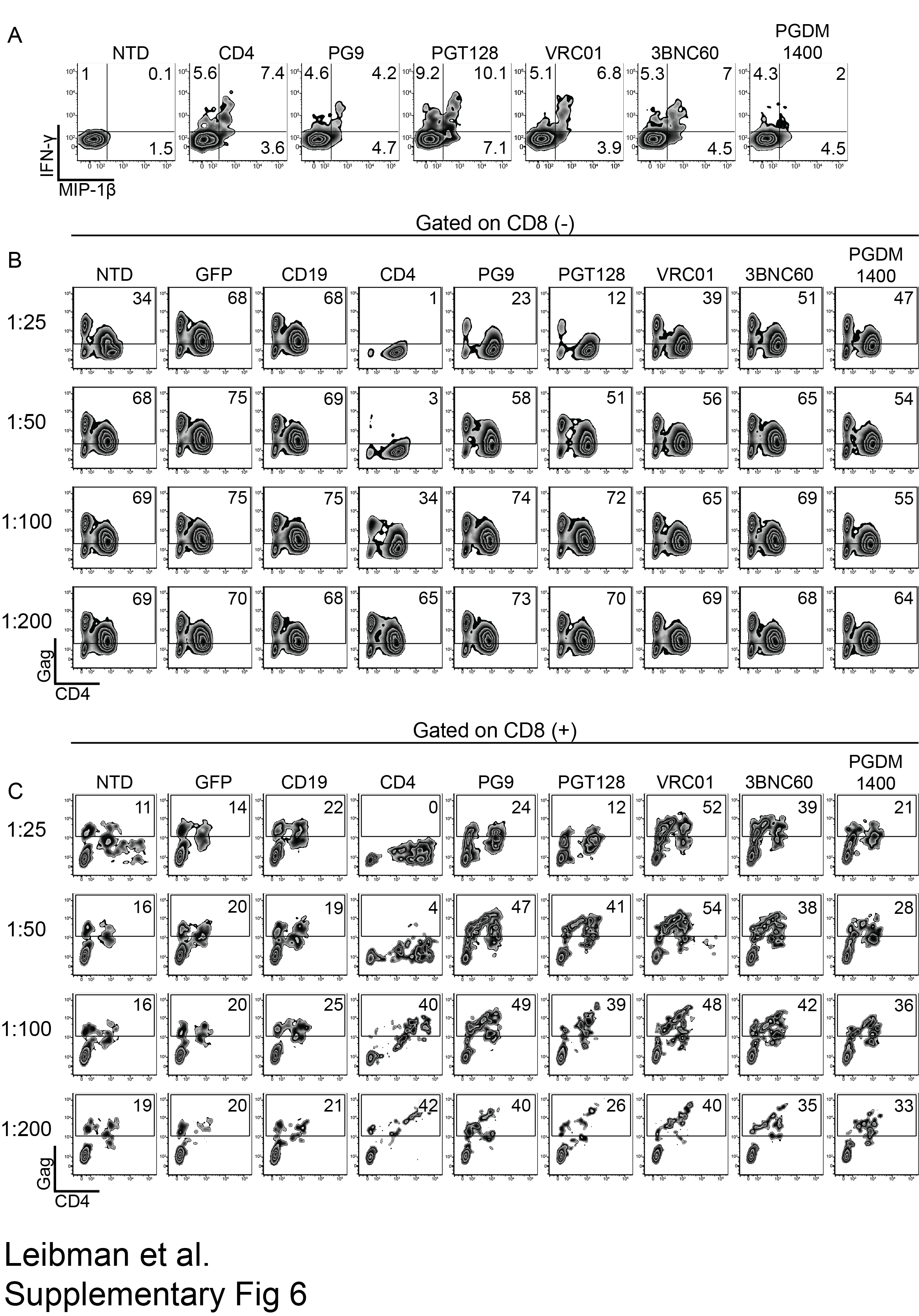

Supplement: S6 Fig — (A) Primary human CD8 T cells were activated either left NTD or transduced with the indicated CAR vectors. Two weeks post activation, the CD8 T cells were co-cultured for 6 hours at a 1:1 ratio with K562 cells expressing HIV-1 YU2 GP160, and intracellular IFNγ and MIP-1β production was measured. Transduction efficiencies were normalized to 60% prior to co-culture. (B) Using the experimental design summarized in Fig 1, the HIV-specific CARs were tested for their ability to control HIV-1 replication in primary human CD4 T cells. NTD, GFP transduced, and CD19-zeta CAR transduced CD8 T cell treatments were included as controls. After 6 days of co-culture, intracellular Gag and CD4 staining is shown for CD8 negative T cells. (C) Shows gating on the CD8 positive cells. (PNG) [file ppat.1006613.s006.png]

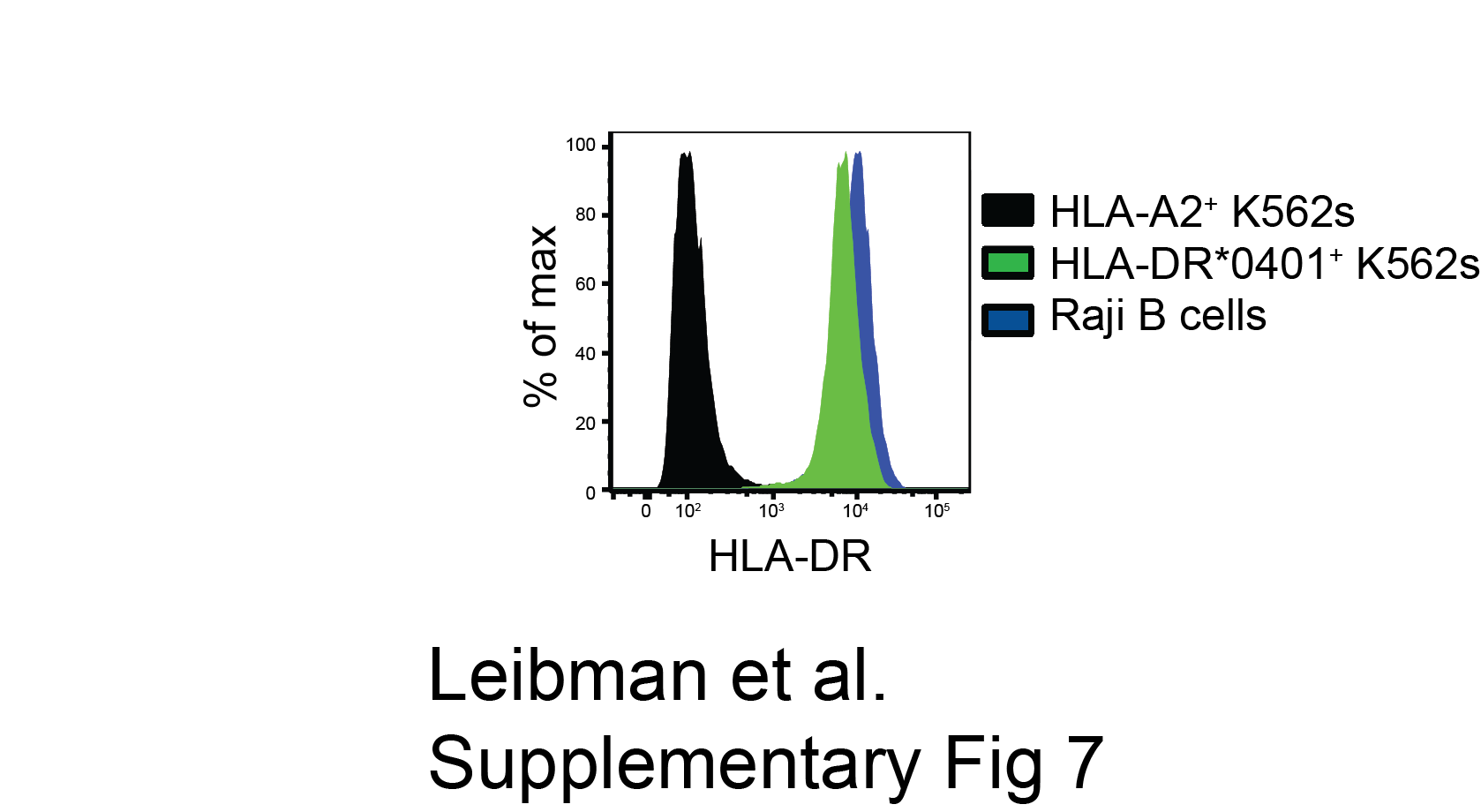

Supplement: S7 Fig — K562 cells transduced with vectors encoding the HLA-DR*0401 α and β chains and single-clone sorted on high expressing cells, were stained for HLA-DR expression along with K562 control cells that had been transduced with HLA-A2, and the MHC class II highly expressing Raji B cells. (PNG) [file ppat.1006613.s007.png]

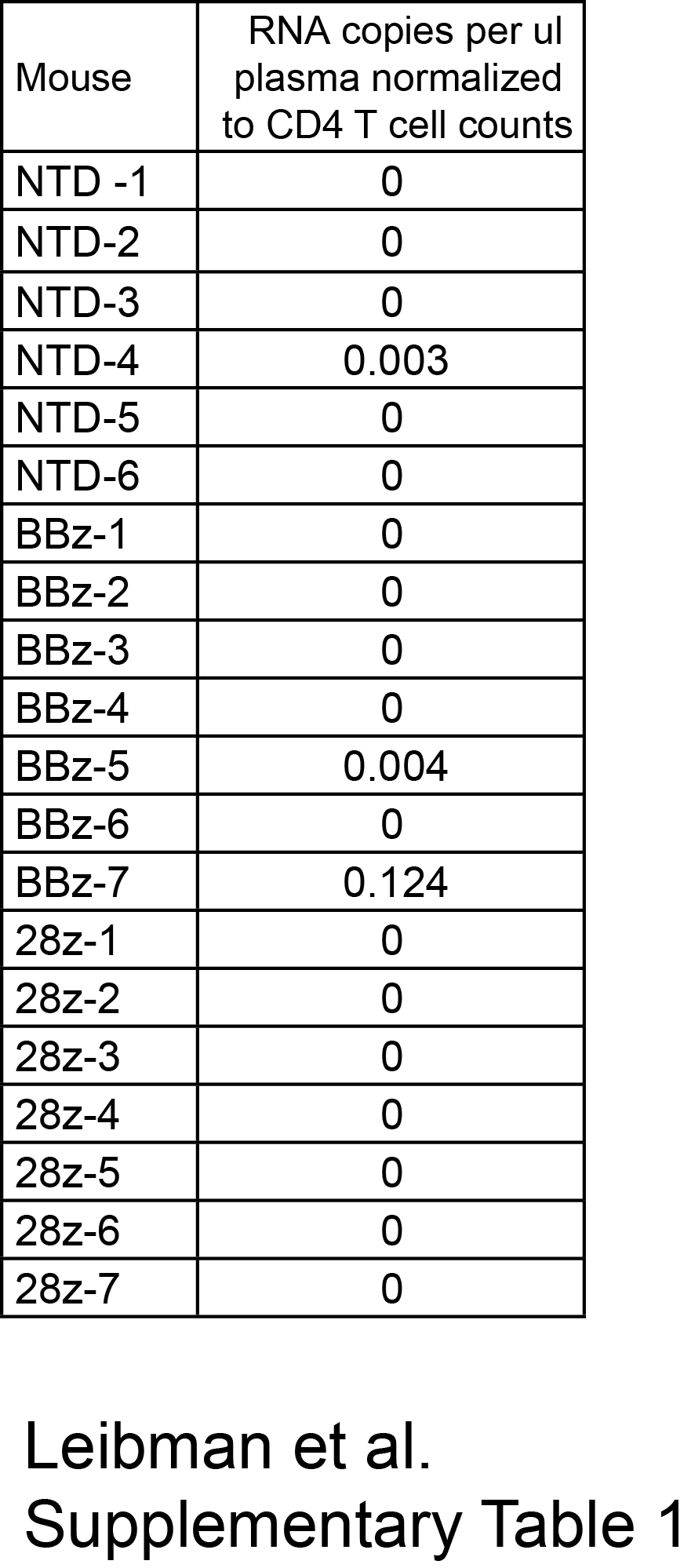

Supplement: S1 Table — Mice were injected with 1 million HIV-infected CD4 T cells (See S8 Fig for timeline) and given daily intraperitoneal injections (200mg/kg) of the reverse transcriptase inhibitor nucleotide analog tenofovir disoproxil fumarate (TDF) for 3 days and then bled for viral load detection. HIV RNA copies per μl plasma were determined by qPCR and normalized to CD4 T cell counts. (PNG) [file ppat.1006613.s020.png]
