# Supplementary material for: Supraphysiologic control over HIV-1 replication mediated by CD8 T cells expressing a re-engineered CD4-based chimeric antigen receptor
Source: PLoS Pathog. 2017 Oct 12;13(10):e1006613. doi: 10.1371/journal.ppat.1006613 (PMC5638568; doi:10.1371/journal.ppat.1006613)
Supplement: S19 Fig — (DOCX) [file ppat.1006613.s019.docx]

**Optimized CD4 CARs:**

**CD4 CD28 CD3-zeta sequence:**

MNRGVPFRHLLLVLQLALLPAATQGKKVVLGKKGDTVELTCTASQKKSIQFHWKNSNQIKILGNQGSFLTKGPSKLNDRADSRRSLWDQGNFPLIIKNLKIEDSDTYICEVEDQKEEVQLLVFGLTANSDTHLLQGQSLTLTLESPPGSSPSVQCRSPRGKNIQGGKTLSVSQLELQDSGTWTCTVLQNQKKVEFKIDIVVLAFQKASSIVYKKEGEQVEFSFPLAFTVEKLTGSGELWWQAERASSSKSWITFDLKNKEVSVKRVTQDPKLQMGKKLPLHLTLPQALPQYAGSGNLTLALEAKTGKLHQEVNLVVMRATQLQKNLTCEVWGPTSPKLMLSLKLENKEAKVSKREKAVWVLNPEAGMWQCLLSDSGQVLLESNIKVLPTWSTPVQPSGTTTPAPRPPTPAPTIASQPLSLRPEACRPAAGGAVHTRGLDFACDFWVLVVVGGVLACYSLLVTVAFIIFWVRSKRSRLLHSDYMNMTPRRPGPTRKHYQPYAPPRDFAAYRSIDRVKFSRSADAPAYQQGQNQLYNELNLGRREEYDVLDKRRGRDPEMGGKPRRKNPQEGLYNELQKDKMAEAYSEIGMKGERRRGKGHDGLYQGLSTATKDTYDALHMQALPPR

Yellow – CD4 EC domain

Turqoise – CD8α hinge

Red – CD28 TM and ICD

Green - CD3 zeta

**CD4 4-1BB CD3-zeta sequence:**

MNRGVPFRHLLLVLQLALLPAATQGKKVVLGKKGDTVELTCTASQKKSIQFHWKNSNQIKILGNQGSFLTKGPSKLNDRADSRRSLWDQGNFPLIIKNLKIEDSDTYICEVEDQKEEVQLLVFGLTANSDTHLLQGQSLTLTLESPPGSSPSVQCRSPRGKNIQGGKTLSVSQLELQDSGTWTCTVLQNQKKVEFKIDIVVLAFQKASSIVYKKEGEQVEFSFPLAFTVEKLTGSGELWWQAERASSSKSWITFDLKNKEVSVKRVTQDPKLQMGKKLPLHLTLPQALPQYAGSGNLTLALEAKTGKLHQEVNLVVMRATQLQKNLTCEVWGPTSPKLMLSLKLENKEAKVSKREKAVWVLNPEAGMWQCLLSDSGQVLLESNIKVLPTWSTPVQPSGTTTPAPRPPTPAPTIASQPLSLRPEACRPAAGGAVHTRGLDFACDIYIWAPLAGTCGVLLLSLVITLYCKRGRKKLLYIFKQPFMRPVQTTQEEDGCSCRFPEEEEGGCELRVKFSRSADAPAYQQGQNQLYNELNLGRREEYDVLDKRRGRDPEMGGKPRRKNPQEGLYNELQKDKMAEAYSEIGMKGERRRGKGHDGLYQGLSTATKDTYDALHMQALPPR

Yellow – CD4 EC domain

Turqoise – CD8α hinge

Pink - CD8α TM

Red – 4-1BB ICD

Green - CD3 zeta

**Antibody Based CARs**

**PG9 CD3-zeta sequence:**

QRLVESGGGVVQPGSSLRLSCAASGFDFSRQGMHWVRQAPGQGLEWVAFIKYDGSEKYHADSVWGRLSISRDNSKDTLYLQMNSLRVEDTATYFCVREAGGPDYRNGYNYYDFYDGYYNYHYMDVWGKGTTVTVSSGGGGSGGGGSGGGGSQSALTQPASVSGSPGQSITISCNGTSNDVGGYESVSWYQQHPGKAPKVVIYDVSKRPSGVSNRFSGSKSGNTASLTISGLQAEDEGDYYCKSLTSTRRRVFGTGTKLTVLSGTTTPAPRPPTPAPTIASQPLSLRPEACRPAAGGAVHTRGLDFACDIYIWAPLAGTCGVLLLSLVITLYCRVKFSRSADAPAYQQGQNQLYNELNLGRREEYDVLDKRRGRDPEMGGKPRRKNPQEGLYNELQKDKMAEAYSEIGMKGERRRGKGHDGLYQGLSTATKDTYDALHMQALPPR

**PGT128 CD3-zeta sequence:**

QSALTQPPSASGSPGQSITISCTGTSNNFVSWYQQHAGKAPKLVIYDVNKRPSGVPDRFSGSKSGNTASLTVSGLQTDDEAVYYCGSLVGNWDVIFGGGTKLTVLGGSSRSSSSGGGGSGGGGQPQLQESGPTLVEASETLSLTCAVSGDSTAACNSFWGWVRQPPGKGLEWVGSLSHCASYWNRGWTYHNPSLKSRLTLALDTPKNLVFLKLNSVTAADTATYYCARFGGEVLRYTDWPKPAWVDLWGRGTLVTVSSSGTTTPAPRPPTPAPTIASQPLSLRPEACRPAAGGAVHTRGLDFACDIYIWAPLAGTCGVLLLSLVITLYCRVKFSRSADAPAYQQGQNQLYNELNLGRREEYDVLDKRRGRDPEMGGKPRRKNPQEGLYNELQKDKMAEAYSEIGMKGERRRGKGHDGLYQGLSTATKDTYDALHMQALPPRR

**VRC01 CD3-zeta sequence:**

EIVLTQSPGTLSLSPGETAIISCRTSQYGSLAWYQQRPGQAPRLVIYSGSTRAAGIPDRFSGSRWGPDYNLTISNLESGDFGVYYCQQYEFFGQGTKVQVDIKRGGSSRSSSSGGGGSGGGGQVQLVQSGGQMKKPGESMRISCRASGYEFIDCTLNWIRLAPGKRPEWMGWLKPRGGAVNYARPLQGRVTMTRDVYSDTAFLELRSLTVDDTAVYFCTRGKNCDYNWDFEHWGRGTPVIVSSSGTTTPAPRPPTPAPTIASQPLSLRPEACRPAAGGAVHTRGLDFACDIYIWAPLAGTCGVLLLSLVITLYCRVKFSRSADAPAYQQGQNQLYNELNLGRREEYDVLDKRRGRDPEMGGKPRRKNPQEGLYNELQKDKMAEAYSEIGMKGERRRGKGHDGLYQGLSTATKDTYDALHMQALPPR

**3BNC60 CD3-zeta sequence:**

DIQMTQSPSSLSARVGDTVTITCQANGYLNWYQQRRGKAPKLLIYDGSKLERGVPARFSGRRWGQEYNLTINNLQPEDVATYFCQVYEFIVPGTRLDLKGGSSRSSSSGGGGSGGGGQVHLSQSGAAVTKPGASVRVSCEASGYKISDHFIHWWRQAPGQGLQWVGWINPKTGQPNNPRQFQGRVSLTRQASWDFDTYSFYMDLKAVRSDDTAIYFCARQRSDFWDFDVWGSGTQVTVSSSGTTTPAPRPPTPAPTIASQPLSLRPEACRPAAGGAVHTRGLDFACDIYIWAPLAGTCGVLLLSLVITLYCRVKFSRSADAPAYQQGQNQLYNELNLGRREEYDVLDKRRGRDPEMGGKPRRKNPQEGLYNELQKDKMAEAYSEIGMKGERRRGKGHDGLYQGLSTATKDTYDALHMQALPPR

**PGDM1400 CD3-zeta sequence:**

DFVLTQSPHSLSVTPGESASISCKSSHSLIHGDRNNYLAWYVQKPGRSPQLLIYLASSRASGVPDRFSGSGSDKDFTLKISRVETEDVGTYYCMQGRESPWTFGQGTKVDIKGGSSRSSSSGGGGSGGGGQAQLVQSGPEVRKPGTSVKVSCKAPGNTLKTYDLHWVRSVPGQGLQWMGWISHEGDKKVIVERFKAKVTIDWDRSTNTAYLQLSGLTSGDTAVYYCAKGSKHRLRDYALYDDDGALNWAVDVDYLSNLEFWGQGTAVTVSSSGTTTPAPRPPTPAPTIASQPLSLRPEACRPAAGGAVHTRGLDFACDIYIWAPLAGTCGVLLLSLVITLYCRVKFSRSADAPAYQQGQNQLYNELNLGRREEYDVLDKRRGRDPEMGGKPRRKNPQEGLYNELQKDKMAEAYSEIGMKGERRRGKGHDGLYQGLSTATKDTYDALHMQALPPR

Yellow – ScFv EC domain

Turqoise – CD8α hinge

Pink - CD8α TM

Green - CD3 zeta

**Supplementary Figure 19: Annotated Sequence Files for Optimized CD4 CARs and Antibody Based CARs**
